# Kaiso (*ZBTB33*) subcellular partitioning functionally links LC3A/B, the tumor microenvironment, and breast cancer survival

**DOI:** 10.1038/s42003-021-01651-y

**Published:** 2021-02-01

**Authors:** Sandeep K. Singhal, Jung S. Byun, Samson Park, Tingfen Yan, Ryan Yancey, Ambar Caban, Sara Gil Hernandez, Stephen M. Hewitt, Heike Boisvert, Stephanie Hennek, Mark Bobrow, Md Shakir Uddin Ahmed, Jason White, Clayton Yates, Andrew Aukerman, Rami Vanguri, Rohan Bareja, Romina Lenci, Paula Lucia Farré, Adriana De Siervi, Anna María Nápoles, Nasreen Vohra, Kevin Gardner

**Affiliations:** 1 Department of Pathology, School of Medicine and Health Sciences, Department of Computer Science, School of Electrical Engineering and Computer Science, University of North Dakota, Grand Forks, ND USA; 2 Division of Intramural Research, National Institutes of Minority Health and Health Disparities, National Institutes of Health, Bethesda, MD USA; 3 Department of Pathology and Cell Biology, Columbia University Irvine Medical Center, New York, NY USA; 4 Laboratory of Pathology, Centers for Cancer Research, National Cancer Institute, National Institutes of Health, Bethesda, MD USA; 5 Ultivue, Inc, Cambridge, MA USA; 6 Department of Biology and Center for Cancer Research, Tuskegee University, Tuskegee, Al USA; 7 Department Computer Science Department, Columbia University, New York, NY USA; 8 Laboratorio de Oncologıa Molecular y Nuevos Blancos Terapeuticos, Instituto de Biologıa y Medicina Experimental (IBYME), CONICET, Buenos Aires, Argentina; 9 Brody School of Medicine, East Carolina University, Greenville, NC USA; 10 Present Address: National Institutes of Genome Research, National Institutes of Health, Bethesda, MD USA

**Keywords:** Prognostic markers, Breast cancer

## Abstract

The use of digital pathology for the histomorphologic profiling of pathological specimens is expanding the precision and specificity of quantitative tissue analysis at an unprecedented scale; thus, enabling the discovery of new and functionally relevant histological features of both predictive and prognostic significance. In this study, we apply quantitative automated image processing and computational methods to profile the subcellular distribution of the multi-functional transcriptional regulator, Kaiso (*ZBTB33*), in the tumors of a large racially diverse breast cancer cohort from a designated health disparities region in the United States. Multiplex multivariate analysis of the association of Kaiso’s subcellular distribution with other breast cancer biomarkers reveals novel functional and predictive linkages between Kaiso and the autophagy-related proteins, LC3A/B, that are associated with features of the tumor immune microenvironment, survival, and race. These findings identify effective modalities of Kaiso biomarker assessment and uncover unanticipated insights into Kaiso’s role in breast cancer progression.

## Abstract (web summary)

Through automated image analysis, Singhal et al quantify nuclear versus cytoplasmic distribution of the Kaiso transcription factor in breast cancer patient tissue. They find that Kaiso distribution correlates with breast cancer subtype and overall survival, and discover a link between cytoplasmic Kaiso and autophagy marker LC3.

## Introduction

Last year in the United States there were over 260,000 new cases of invasive breast cancer and, by the year’s end, over 40,000 women diagnosed with breast cancer died from their disease^1^. Breast cancer is a very heterogeneous disease with various subtypes of diagnostic and prognostic significance that differ in distribution by both age and race^2^. A major tool in the diagnosis, management, and prevention of breast cancer is the identification and characterization of biomarkers that will predict survival, and guide treatment decisions with higher precision^3^. Traditional visual assessment of histopathological images in combination with antibody-based biomarker profiling has been the standard of practice. However, the recent application of algorithms for digital image analysis to augment image characterization and quantitative assessment has broadened the utility, application, and accuracy of antibody-based characterization, thus providing new insight into the functional roles of specific protein biomarkers, and spawning a new age of diagnostic, prognostic, and therapeutic innovation^4–7^. Here we apply multiple algorithms for digital image analysis to perform an antibody-based assessment of patient breast cancer tissues that quantitatively profiles the subcellular distribution of Kaiso (*ZBTB33)*, a functional protein biomarker previously shown to be elevated in cancers of the breast, prostate, pancreas, and colon^8–10^.

Kaiso was originally identified as a transcription factor and member of the BTB/POZ (Broad complex, Tramtrak, Bric à Brac/Poxvirus zinc finger) subfamily of zinc finger proteins^8,10^. It contains a bimodal DNA-binding domain that recognizes both sequence-specific consensus sites and methylated CpG nucleotides through 3 C-terminal DNA-binding Kruppel-like CH2 zinc fingers^8,10^. In its nuclear capacity, it has been shown to drive transcriptional programs that increase breast cancer growth and metastasis^8,10^. Subsequent studies revealed that it binds to a variety of sequence motifs throughout the nucleus, the majority of which are unmethylated and located at loci with open chromatin^8,11^. Because of its role in transcriptional regulation, Kaiso has been predominantly investigated as a nuclear protein and multiple studies have shown that its nuclear accumulation is dynamically regulated by association with p120 (CTNND1)^12^, a catenin family member that regulates membrane-bound E-cadherin cell adhesion assemblies^8^. Although several studies have noted that the cytoplasmic localization of Kaiso is a common feature in human tissues and tumors^9^, the major emphasis placed on Kaiso, as a prognostic biomarker, has focused on its nuclear accumulation. In fact, the nuclear accumulation of Kaiso has been shown, in multiple studies, to be a predominant feature of more aggressive forms of breast cancer, including triple-negative breast cancer^13,14^. Moreover, in some studies, nuclear Kaiso was shown to be more predictive of poor survival in women of African heritage diagnosed with TNBC^14,15^. Nonetheless, there have been multiple reported observations of cytoplasmic or “non-nuclear” accumulation of Kaiso, implying potential functional roles for Kaiso outside of the nucleus^9,16,17^. These include interaction with centrosomes, assembly with RhoH and p120 at actin-containing cell protrusions, and regulation of Kaiso subcellular distribution by the EGF-1^9,18,19^.

In this study, we leverage the analytic precision of automated image analysis algorithms to quantitatively profile the compartment-specific (nuclear versus cytoplasmic) distribution of Kaiso in a racially diverse cohort of breast cancer patients residing in a health disparities region of rural East North Carolina. These findings reveal that both nuclear and cytoplasmic Kaiso are associated with breast cancer outcome and each are independent predictors of overall breast cancer survival. Furthermore, compartment-specific profiling of Kaiso with multiple prognostic breast cancer biomarkers reveal new functional correlations that link the specific subcellular distribution of Kaiso with (1) the autophagy-related factor LC3A/B; (2) cellular phenotypes within the tumor immune microenvironment; and (3) overall breast cancer survival.

## Results

### The subcellular distribution of Kaiso is differentially correlated with breast cancer subtype and overall survival from breast cancer

**Fig. 1:**
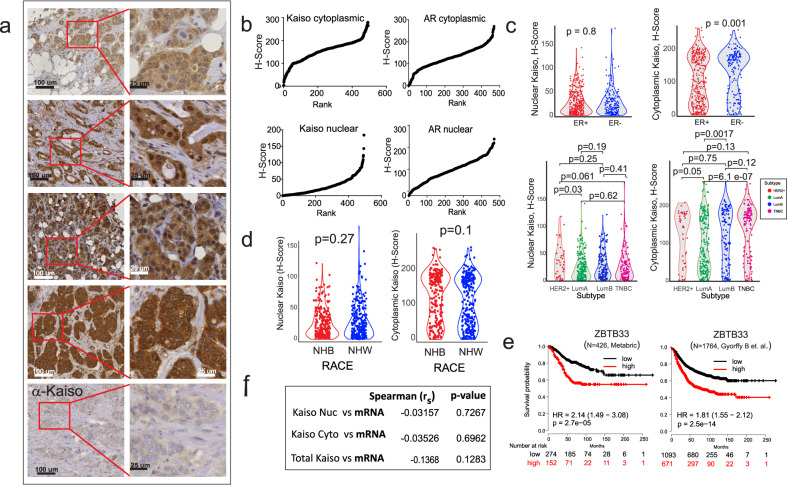
Profiles of the subcellular distribution of Kaiso (*ZBTB33*) show that nuclear and cytoplasmic Kaiso are differentially correlated with breast cancer subtype and hormone status. **a** Representative subcellular patterns of Kaiso expression in breast cancer tissues detected by anti -Kaiso immunohistochemical staining. Shown is a range of high (upper panels) versus low (bottom panel) nuclear and cytoplasmic protein expression. **b** Comparison of the distribution of digitally determined H-scores for nuclear versus cytoplasmic Kaiso enrichment. The difference in distribution is shown in contrast to the similarities in the distribution of nuclear versus cytoplasmic androgen receptor (AR). **c** Cytoplasmic Kaiso is differentially enriched in ER^−^ breast cancers compared to nuclear Kaiso and is significantly enriched in the more aggressive breast cancer subtypes, LumB, HER2^+^, and TNBC. **d** Nuclear and cytoplasmic levels of Kaiso (*H*-score) do not show significant differences (*t*-test) based on race (also see Supplementary Fig. 1). **e** Kaiso (*ZBTB33*) mRNA abundance (median) is predictive of poor overall breast cancer survival as demonstrated in two independent publicly available breast cancer cohorts^88^. **f** Comparison of nuclear and cytoplasmic levels of Kaiso in breast cancer patients to levels detected by RNA-seq in (*N* = 131) patients demonstrates that Kaiso (*ZBTB33*) mRNA levels do not correlate with either nuclear or cytoplasmic levels of Kaiso. LumA Luminal A, LumB Luminal B, HER2^+^ human epidermal growth factor receptor 2 positive, TNBC triple-negative BC, ER estrogen-receptor status, NHW non-Hispanic White, NHB non-Hispanic White.

The protein expression of Kaiso was spatially profiled by automated analysis^4,6^ of immunohistochemically stained tissue microarrays (TMAs) (Fig. 1), containing 555 tumors from a cohort of racially diverse breast cancer patients (see Supplementary Fig. 1) residing in a designated health disparities catchment area of East North Carolina (median follow-up 8.5 years)^6^. By this analysis, staining intensity in the nucleus or cytoplasm of each cell in the annotated tumor regions is assigned one of four scores: from negative staining (0) to weak staining (+1), moderate staining (2+) or strong staining (3+). The percent of cells in the annotated regions demonstrating one of the 4 different intensities are then aggregated to derive an *H*-score (*H*-score= 3 × [%3+] + 2 × [%2+] + 1 [%1+]), thus generating a continuous score from 0 to 300^4,6^. The subcellular distribution of the staining intensity differs across many of the tumors, revealing various patterns of enrichment in the nucleus, cytoplasm, or both. (Fig. 1a). Notably, the cell segmentation algorithm-based quantitative profiling^4,6^ of Kaiso subcellular distribution reveals distinct differences in the cytoplasmic versus nuclear Kaiso patterns of distribution when examined in rank order (Fig. 1b, left). This Kaiso-specific difference in subcellular distributions is contrasted by the significant similarity in the nuclear and cytoplasmic distribution of a typical nuclear antigen that shuttles between the cytoplasm and nucleus like the androgen receptor (AR) (Fig. 1b, right), as well as the lower correlation between nuclear and cytoplasmic Kaiso, compared to AR (Supplementary Fig. 2). These stark differences suggest that the mechanisms governing the nuclear versus cytoplasmic localization of Kaiso are far more dynamic and complex than the androgen receptor whose intensity distribution in the nucleus reflects a more passive ligand-mediated nuclear-to-cytoplasmic distribution.

Unlike nuclear Kaiso, the levels of cytoplasmic Kaiso in this cohort were significantly different based on hormone receptor (ER) status and breast cancer subtype, where the cytoplasmic levels of Kaiso were distinctly higher in the subtypes of breast cancer known to be more aggressive, including triple-negative breast cancer (TNBC), human epidermal growth factor receptor 2 positive (HER2^+^), and Luminal B (LumB) (Fig. 1c). Although the levels of nuclear Kaiso have been previously reported to be higher in patients of African heritage^15,20^, we did not detect such differences in the current cohort although a minor trend for preferential distribution of high levels of cytoplasmic Kaiso in patients of African, compared to European descent, was observed (Fig. 1d). Interestingly, although the mRNA levels of Kaiso are highly predictive of poor breast cancer survival, as demonstrated in publicly available data sets (Fig. 1e); a direct comparison of available RNA-seq expression data, within a subset of this cohort (*N* = 134), demonstrates very little correlation between Kaiso mRNA and either nuclear Kaiso, cytoplasmic Kaiso, or their combined total (Fig. 1f).

**Fig. 2:**
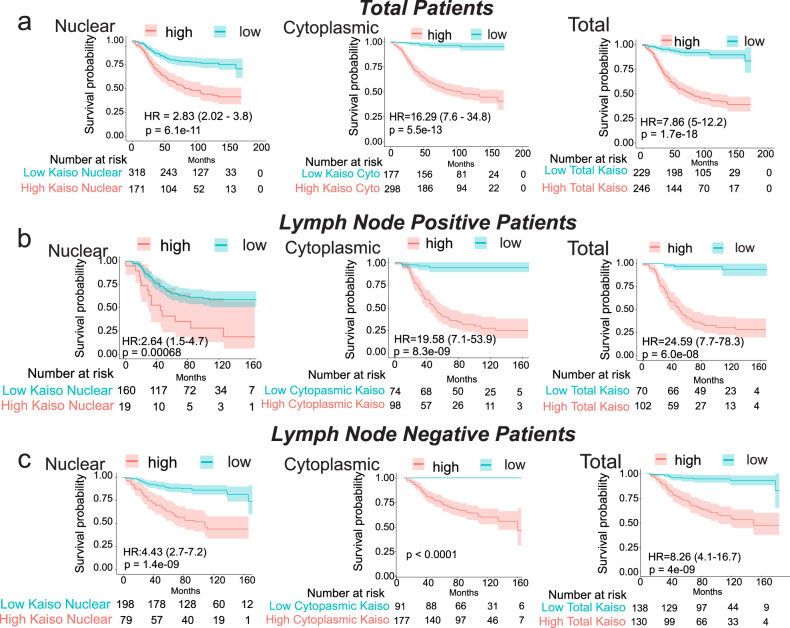
Both nuclear Kaiso and cytoplasmic Kaiso are predictive of poor breast cancer survival. **a** Analysis of the association between subcellular Kaiso distribution and survival demonstrates that high cytoplasmic Kaiso is much more predictive of poor survival compared to nuclear Kaiso. Nuclear, cytoplasmic, and total Kaiso *H*-scores predict survival in both **b** high risk (lymph node-positive) and **c** low risk (lymph node-negative) breast cancer patients, where total Kaiso score is most predictive of survival in both low and high-risk breast cancer patients. (HR could not be calculated for cytoplasmic Kaiso in low-risk patients because no deaths were recorded in that risk group). NHW non-Hispanic white; NHB non-Hispanic black. Optimized cut-offs were determined by the method of maximally selected rank statistics (Supplementary Fig. 3 and Supplementary Table 2).

Nuclear Kaiso, cytoplasmic Kaiso, and their combined score, defined as total Kaiso, are highly correlated with poor breast cancer survival (Fig. 2a–c). However, cytoplasmic Kaiso appears to be significantly more predictive with a hazard ratio (HR) of 16.29 (confidence interval (CI): 7.6–34.8; *p*-value 5.3E−13) for cytoplasmic Kaiso compared to HR: 2.83 (CI: 2.02–3.9; *p*-value 6.1E−11) for nuclear Kaiso, and HR: 7.86 (CI: 5.0–12.22; *p*-value 1.7E−18) for total Kaiso (Fig. 2a). These differences are also in good agreement with a non-digitally assisted expert pathologist’s assessment (Supplementary Fig. 4). In the multivariate setting, both nuclear and cytoplasmic Kaiso are independent predictors of overall breast cancer survival (Table 1). Finally, both nuclear and cytoplasmic Kaiso can stratify patients with lymph node-positive (high risk of recurrence) (Fig. 2b) or lymph node-negative breast cancer (low risk of recurrence) (Fig. 2c) into high versus low survival groups. However, in each case, although both nuclear Kaiso and cytoplasmic Kaiso are independent predictors of survival, cytoplasmic Kaiso is consistently more predictive of poor overall survival compared to nuclear Kaiso (Fig. 2a–c). Interestingly, total Kaiso appears to be the best predictor of overall survival in high risk (lymph node-positive) patients, with a hazard ratio of HR: 24.59 compared to HR: 19.58 (cytoplasmic Kaiso) or HR: 2.64 (nuclear Kaiso) (Fig. 2b).

**Table 1:** Univariate and multivariate analysis of the hazard ratio for overall survival associated with patient demographics, subtype, nuclear Kaiso, cytoplasmic Kaiso, and cytoplasmic LC3A/B expression in patient breast cancer samples.

Univariate analysis	HR (95% CI for HR)	*p*-value	Multivariate analysis	HR (95% CI for HR)	*p*-value
AGE	**1.02** (1.01–1.03)	**2.00E−03**	AGE	1.02 (1.00–1.05)	5.28E−02
RACE (Black)	1.18 (0.89–1.56)	2.60E−01	RACE (Black)	0.85 (0.58–1.26)	4.25E−01
Menopause status	0.76 (0.55–1.12)	1.20E−01	Menopause status	0.89 (0.47–1.70)	7.23E−01
BMI	0.10 (0.98–1.00)	8.10E−01	BMI	0.98 (0.96–1.00)	1.00E−01
ER status	**1.76** (1.32–2.35)	**1.30E**−**04**	ER status	1.01 (0.49–2.10)	9.89E−01
Nuclear Kaiso	**1.01** (1.01–1.02)	**2.00E**−**09**	Nuclear Kaiso	**1.01** (1.01–1.02)	**2.29E**−**06**
Cytoplasmic Kaiso	**1.01** (1.02–1.01)	**3.96E**−**10**	Cytoplasmic Kaiso	**1.01** (1.01–1.01)	**2.98E**−**07**
LC3AB	**1.00** (1.00–1.01)	**5.90E**−**05**	LC3AB	1.00 (1.0–1.00)	6.06E−01
Node (positive)	**2.30** (1.69–3.13)	**1.30E**−**07**	Node (positive)	**2.64** (1.79–3.89)	**8.78E**−**07**
Subtype (LumA)			Subtype (LumA)		
*LumB*	1.29 (0.87–1.93)	2.06E−01	*LumB*	1.31 (0.77–2.21)	3.19E−01
*HER2*+	1.54 (0.91–2.62)	1.10E−01	*HER2*+	1.83 (0.76–4.43)	1.81E−01
*TNBC*	2.50 (1.77–3.53)	**2.25E**−**07**	*TNBC*	2.25 (1.0–5.08)	5.20E−02

Referent for Subtype is Luminal A subtype. Referent for menopause status is pre-menopause. Referent for Race is White. Referent for Node status is positive. Significant *p*-values (<0.05) are in bold.

### Cytoplasmic Kaiso reveals a high correlation with TNBC and the autophagy-related protein LC3A/B

**Fig. 3:**
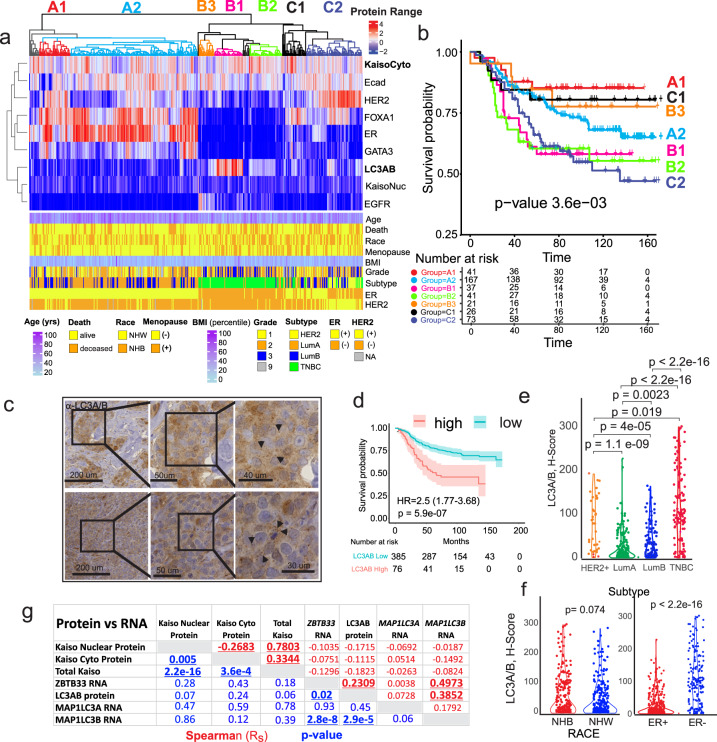
Quantitative comparison of digitally scored functional and predictive biomarker abundance reveals associations between cytoplasmic Kaiso and the autophagy-related antigen, LC3A/B, that correlate with subtype and survival. **a** Unsupervised hierarchical clustering of the nuclear, membrane and cytoplasmic biomarker *H*-scores for each of (*N* = 555) patients is shown in correlation with patient clinicopathologic and demographic attributes (below). **b** Kaplan–Meier survival analysis of specific antigen expression clusters (identified by color code) in **a** demonstrating associations between cytoplasmic Kaiso, LC3A/B expression, and overall survival in TNBC. **c** Representative sample of LC3A/B immune-histochemical staining in breast cancer tissues. Arrowhead indicates subcellular puncta noted in the cytoplasm of multiple sections. **d** Kaplan–Meier survival analysis shows that high LC3A/B cytoplasmic staining is associated with poor overall breast cancer survival. **e** Like cytoplasmic Kaiso, LC3A/B staining is highly correlated with the more aggressive breast cancer subtypes LumB, HER2, and TNBC with the strongest association with TNBC. **f** LC3A/B shows a trend of higher expression in NHB versus NHW patients and is significantly more expressed in patients with ER- breast cancer. **g** Correlation between LC3A/B protein, nuclear Kaiso, cytoplasmic Kaiso, and the RNA levels for LC3A/B, (*MAPL1LC3A, MAPL1LC3B*), and Kaiso (*ZBTB33*) showing the strongest correlation between LC3A/B and *MAPL1L3*B RNA in addition to *ZBTB3*3 RNA and *MAPL1LC3B* RNA. Spearman correlation is shown in red. *p*-value for Spearman correlation is shown in blue.

The high correlation between cytoplasmic Kaiso expression and more aggressive subtypes of breast cancer (Fig. 1c) implies that similar comparisons, between cytoplasmic Kaiso and other biomarkers that stratify aggressive forms of cancer, will provide deeper insights into the role of cytoplasmic Kaiso in poor breast cancer survival. To accomplish this goal, quantitative profiles comparing the relative enrichment of biomarkers recently implicated to be strongly associated with breast cancer progression^21–29^, were analyzed together by robust unsupervised hierarchical clustering (Fig. 3a). Biomarkers investigated included, estrogen-receptor ER^30^, the ER pioneer proteins FOXA1^31–33^ and GATA3^34–36^, HER2 membrane expression^37,38^, the protein-membrane adhesion molecule and tumor suppressor E-cadherin^39–41^, epithelial growth factor receptor EGFR^37,38^, and the autophagy-related factor LC3A/B (*MAP1L3A* and *MAP1L3B*), an autophagy-related biomarker recently implicated to be strongly associated with breast cancer progression^21–29^. A heatmap of clinical and pathological features of patients including survival, tumor subtype, and ER status is provided underneath for a direct comparison of biomarker expression with patient characteristics. Notably, patient clustering by these biomarkers in combination with cytoplasmic Kaiso expression identifies multiple breast cancer groups (A1-C2) with distinct survival differences (Fig. 3b and Supplementary Fig. 5).

Of particular interest is the clustering of cytoplasmic Kaiso with the autophagy marker LC3A/B (Fig. 3a), which, in combination with the other biomarkers, stratifies TNBC patients into 3 different survival subgroups (B1, B2, and B3). The LC3A/B family of proteins has a major role in a variety of autophagy-related phagocytic and secretory processes including autophagy, phagocytosis, conventional secretion of cytokines, extracellular release of lysozymes, extracellular vesicle (EV) production, unconventional protein secretion, and LC3-dependent EV loading and secretion (LDELS)^42–45^. In each case, lipid conjugated LC3 has a major role in the loading of different cargoes, (organelles, protein, and nucleic acid) into membrane-bound structures destined for secretion or degradation. Collectively these processes are referred to as secretory autophagy^43,44,46^. Furthermore, each of these processes has the potential to influence tumor microenvironment^47^.

Remarkably, the clustering of low cytoplasmic Kaiso and low LC3A/B, in the context of the other biomarkers, identifies a class of TNBC patients that show favorable survival (compare B1 & B2, to B3) (Fig. 3b). Thus, low levels of cytoplasmic Kaiso combined with low levels of the autophagy-related factor LC3A/B, predict favorable survival in breast cancer patients with TNBC, implicating a significant role for this biomarker in tumor progression and survival. Representative LC3A/B IHC staining of patient tumors reveals a heterogeneous staining pattern in the cytoplasm with a mixture of diffuse and punctate cytoplasmic staining typical of LC3A/B^27,48^ (Fig. 3c). Quantitative analysis of the cytoplasmic staining shows that cytoplasmic LC3A/B is predictive of survival (HR: 2.5, CI: 1.77–3.68; *p*-value 5.9e−07) (Fig. 3d and Supplementary Fig. 3). Similar to cytoplasmic Kaiso, LC3A/B is (1) preferentially expressed in more aggressive forms of breast cancer including LumB, HER2, and TNBC (Fig. 3e); and (2) preferentially elevated in ER- compared to ER^+^ tumors with a trend toward higher expression in women of African compared to European ancestry (Fig. 3f). Analysis of the correlation between IHC-based protein expression and RNA expression (Fig. 3g and Supplementary Data 1) reveals very little correlation between Kaiso (*ZBTB33*) mRNA and Kaiso protein. However, there is a modest correlation between *MAP1LC3B* mRNA and LC3A/B with the highest correlation between *ZBTB33*, *MAP1LC3B* mRNA, and LC3A/B protein (Fig. 3g). By univariate survival analysis, LC3A/B expression is significantly predictive of poor survival but loses all significance in the multivariate setting, where only nuclear Kaiso, cytoplasmic Kaiso, and lymph node status are significant independent predictors of outcome (Table 1). This suggests that the survival predictive value of LC3A/B is closely associated with Kaiso protein expression.

### Kaiso is required for the functional activation of LC3A/B

**Fig. 4:**
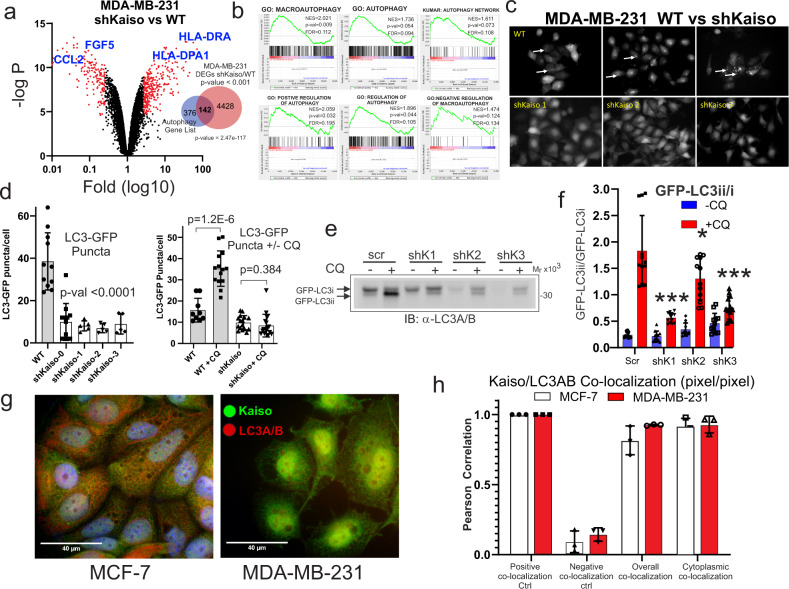
Breast cancer cells depleted of Kaiso are enriched for autophagy terms and show a defect in LC3A/B maturation. **a** Differential gene expression analysis of wild type TNBC cell line MDA-MB-231 versus MDA-MB-231 cells depleted of Kaiso by RNAi shows a significant overlap of differentially expressed genes with an autophagy-related gene list (also see Supplementary Fig. 6, and Supplementary Table 3) (*p*-value for the significance of the overlap is provided by the hypergeometric test). **b** Gene set enrichment analysis shows significant enrichment for autophagy terms in MDA-MB-231 cells that differentially express Kaiso (Supplementary Data 4). **c** Analysis of autophagocytic flux in WT versus MDA-MB-231 depleted of Kaiso by RNAi reveal a significant defect in autophagy demonstrated by decreased autophagocytic puncta formation in cells depleted of Kaiso. **d** Quantitative GFP-LC3 immunofluorescent analysis of LC3 puncta formation (average puncta per cell before (left) and after (right) addition of chloroquine in MDA-MB-231 cells expressing GFP-LC3). **e** Immunoblot analysis of LC3A/B lipidation (GFP-LC3ii formation) in MDA-MB-231 transfected with scramble control RNAi versus 3 different short hairpins against Kaiso. **f** Quantitative densitometer scan (*N* = 4 biological replicates each with *N* = 3 technical replicates). (*) = *p*-value < 0.05, (***) = *p*-value < 0.001 (*t*-test) for each unique Kaiso RNAi hairpin. **g** Immunofluorescent colocalization of Kaiso (green) and LC3A/B (red) in MCF-7 and MDA-MB-231 cells. **h** Quantitative profiling of Kaiso and LC3A/B colocalization in both the cytoplasm and nucleus (overall) of MCF-7 and MDA-MB-231 cells.

Given the close correlation between Kaiso and the LC3A/B autophagy-related proteins, we sought to further establish this relationship by employing a “bedside-to-bench” approach utilizing the human triple-negative breast cancer cell line, MDA-MB-231 depleted of Kaiso by RNA interference (RNAi) (Fig. 4a and Supplementary Fig. 6). The analysis of genes that are significantly differentially expressed (*p*-values < 0.001) in WT versus Kaiso-depleted cells reveals a large overlap (142 of 518) with a list of autophagy-associated genes compiled from the MSigDbase and the Autophagy Database^49,50^ (Fig. 4a) (also see Supplementary Data 2). This overlap is supported further by GSEA revealing a large and substantial enrichment in six (6) different autophagy-related gene sets (Fig. 4b) (also see Supplementary Data 3).

A hallmark feature of autophagy-related processes is the conjugation of LC3A/B to phosphatidylethanolamine (PE) followed by its incorporation into intracellular membrane vesicles within the endocytic vesicle system^44,46,51^. The lipid conjugation and membrane incorporation of LC3 protein enable it to load specific cargo into various endocytic trafficking vesicles including autophagosomes and extracellular vesicles^44–46^. A commonly used method to examine autophagocytic flux is to visualize the incorporation of GFP-labeled LC3A/B into autophagocytic membrane structures that appear as intracytoplasmic puncta (Fig. 4c)^52,53^. As demonstrated in Fig. 4c, MDA-MB-231 cells depleted of Kaiso show a significant absence (*p* < 0.0001) of puncta both in the presence or absence of autophagosome stabilization by the lysosome inhibitor chloroquine (CQ) (Fig. 4d). LC3A/B maturation can be followed by immunoblot analysis through the detection of changes in LC3A/B phosphatidylethanolamine (PE) lipid conjugation by the demonstration of a change in mobility, where the lipid conjugated LC3A/B (LC3ii) migrates with faster mobility than the unconjugated form (LC3i) (Fig. 4e). This difference in LC3 conjugation can be amplified by the inhibition of LC3 lysosomal degradation by chloroquine (CQ) (Fig. 4e, f). As shown in Fig. 4f, compared to the non-targeting short hairpin, the addition of 3 different RNAi short hairpins targeting Kaiso, results in a significant reduction of GFP-LC3 conjugation. Similar results are observed with the ER^+^ cell line MCF-7 (Supplementary Fig. 7). Finally, Kaiso and LC3A/B immunofluorescence show significant colocalization in both ER^+^ (MCF-7) and TNBC (MDA-MB-231) cell lines in both the cytoplasm and nucleus (Fig. 4g, left and right, respectively), with quantitative profiling in Fig. 4h (also see Supplementary Fig. 8).

### Kaiso and LC3A/B show significant colocalization in patient tumors

**Fig. 5:**
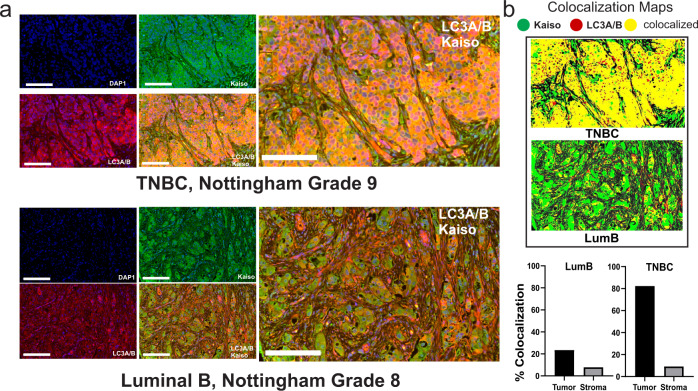
Kaiso and LC3A/B show extensive colocalization in both the nucleus and cytoplasm of patient tumors. **a** Immunofluorescent staining of Kaiso (green) and LC3A/B (red) in TNBC breast cancer (upper panel) and Luminal B breast cancer (lower panel). **b** Colocalization maps showing relative colocalization of Kaiso and LC3A/B in patient tumors.

Consistent with the observations in breast cancer cell lines, there is also significant cytoplasmic colocalization of Kaiso and LC3A/B in patient tumors with substantial variation by subtype (Fig. 5a, b) Thus, consistent with the co-enrichment of cytoplasmic Kaiso with LC3A/B in patient samples (Fig. 3a) and their colocalization in breast cancer cell lines (Fig. 4g), both proteins also show a substantial colocalization in patient tumors that varies by subtype (Fig. 5a, b).

### Patients stratified by nuclear and cytoplasmic Kaiso are variably enriched in cellular stress and immune response pathways and differentially predict the overall outcome based on genetic ancestry

**Fig. 6:**
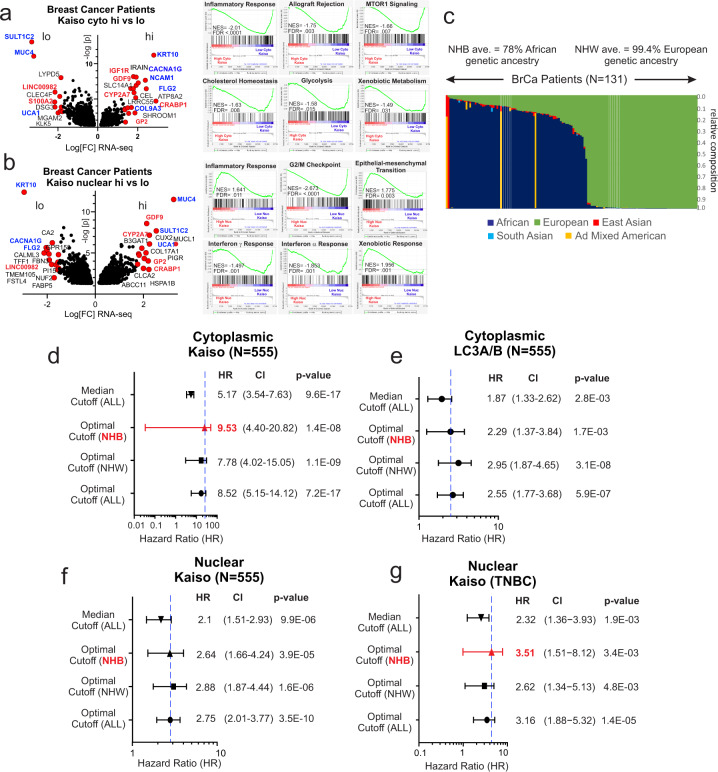
Patients stratified by nuclear and cytoplasmic Kaiso are variably enriched in cell stress and immune response pathways and differentially predict survival based on genetic ancestry. **a** Volcano plot of differential gene expression of patients stratified by nuclear Kaiso. (Right) Gene set enrichment analysis of patients stratified by cytoplasmic Kaiso (Supplementary Data 5 and 6). **b** Volcano plot of differential gene expression of patients stratified by cytoplasmic Kaiso (right) Gene set enrichment analysis of patients stratified by nuclear Kaiso. **c** Population-specific composition of a representative portion (*N* = 131) of the breast cancer cohort, by genetic ancestry-based ancestry informative markers extracted from the RNA-seq data. Indicated colors reflect the percent admixture of each genetic population. **d** Forest plot analysis of overall survival hazard-based optimized cut-off for cytoplasmic Kaiso for the total population, NHW patients, NHB patients, and median cut-off for the total population. **e** Forest plot analysis of overall survival hazard-based optimized cut-off for LC3A/B in the total population, NHW patients, NHB patients, and the median cut-off for the total population. **f** Forest plot analysis of overall survival hazard based on optimized cut-off for nuclear Kaiso in the total population, NHW patients, NHB patients, and the median cut-off for the total population. **g** Forest plot analysis of overall survival hazard based on optimized cut-off for nuclear Kaiso in TNBC patients using optimized cut-off for NHW patients, NHB patients, and the median cut-off for the total TNBC population.

A comparison of the gene expression enrichment patterns of patients stratified by nuclear versus cytoplasmic Kaiso shows concordant (red) and discordant (blue) expression of genes linked to cellular differentiation, metabolism, immune modulation, and cell-microenvironment interactions (Fig. 6a, b). This observation is corroborated by GSEA profiles revealing opposing enrichment for inflammatory response pathways in nuclear versus cytoplasmic Kaiso (Fig. 6a, b, right) with notable negative enrichment for allograft rejection pathways in patients over-expressing cytoplasmic Kaiso (also see Supplementary Data 4 and 5).

As discussed earlier, nuclear Kaiso expression has been linked previously to racial differences in breast cancer outcome where nuclear Kaiso was found to be higher and more predictive of poor outcome in women of African Heritage (non-Hispanic black (NHB)) diagnosed with TNBC compared to their European (non-Hispanic white (NHW)) counterparts^8,14,15^. Most notably this distinction seemed to be greater depending on the degree of African ancestry^15^. To profile the degree of racial admixture in our study cohort, ancestral informative markers (AIMs) were extracted from patient (*N* = 131) tumor RNA-seq data (>23% of the study cohort)^54,55^. Each patient in this group was then assigned a percent ancestry based on five genetic populations (African, European, East Asian, South Asian, and Admixed Native American) Fig. (6c). Among the 69 patients that self-identified as NHB, all but 1 had >50% African ancestry. Of the 62 patients that self-identified as NHW, 3 patients showed greater than 80% admixed Native American ancestry (Fig. 6c). Notably, forest plot analysis of the Cox proportional-hazards model for overall survival, optimized by Race, reveals that cytoplasmic Kaiso is more predictive of survival in women of African ancestry (Fig. 6d), consistent with prior indications of a differential survival risk based on Kaiso and African genetic background^8,14^. Moreover, even within admixed populations, and consistent with previous reports, there is a greater survival risk associated with nuclear Kaiso in patients of African ancestry diagnosed with TNBC (Fig. 6g). However, neither LC3A/B nor Nuclear Kaiso shows significant racial differences in survival hazard in the total breast cancer cohort (Fig. 6e, f).

### Cytoplasmic Kaiso levels and LC3A/B are associated with an immune-suppressed tumor microenvironment in breast cancer tissues

**Fig. 7:**
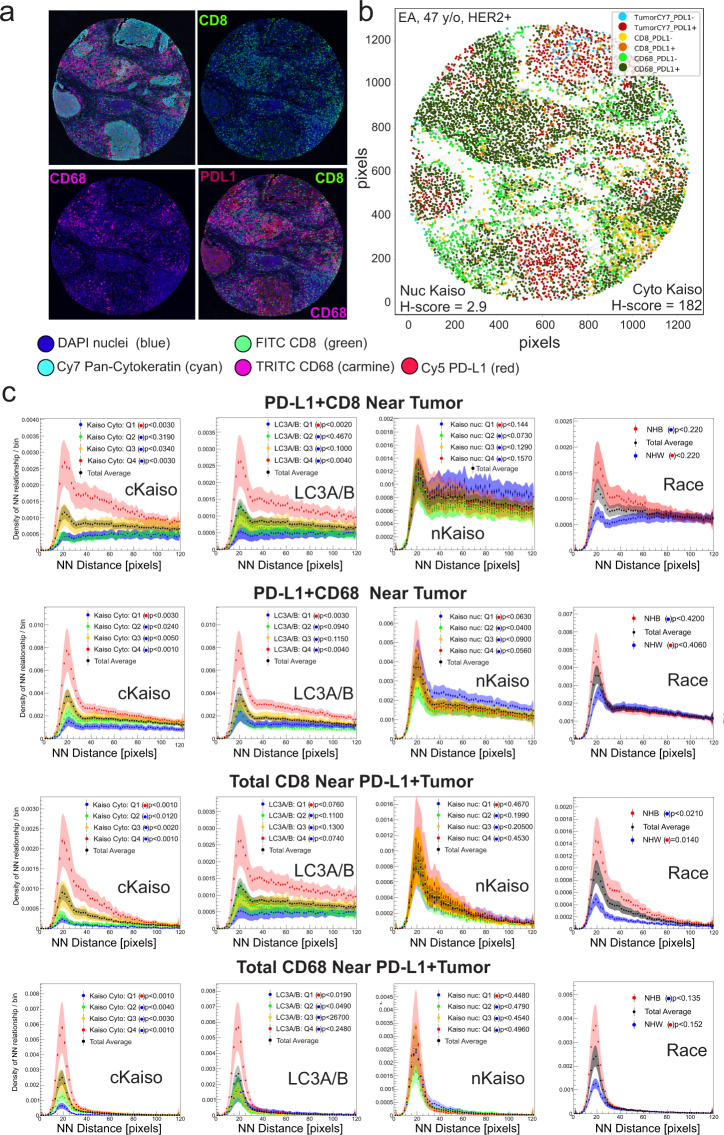
Elevated cytoplasmic Kaiso, LC3A/B, and race are associated with an immune-suppressive tumor microenvironment. **a** Representative multi-spectral quantitative immunofluorescence (mQIF) of the tumor microenvironment of a breast cancer TMA core, stained for pan-cytokeratin (Cy7, cyan); PDL-1 (Cy5, red); CD8 (FITC, green); CD68 (TRITC, carmine). **b** Coordinate map for nearest-neighbor analysis of tumor and stromal immunophenotypes. **c** Nearest-neighbor analysis showing the frequency distribution of immune cell proximities to tumor-associated with expression quartiles (Q1–Q4) of cytoplasmic Kaiso, LC3A/B, nuclear Kaiso, and race (white versus black).

The immune tumor microenvironment has been found to have a broad prognostic and predictive role in breast cancer^56–61^. With respect to breast cancer racial health disparities, there is a wide consensus supporting a deterministic role for race-based genetic variation in the immune response in influencing racial survival disparities in breast cancer^62–67^. Given the observed influence of elevated levels of cytoplasmic and nuclear Kaiso on immune regulatory pathways (Fig. 6a, b), and the extensive potential role for secretory autophagy in the immune response^43,44,46^, we sought to define the linkage between LC3A/B, the subcellular distribution of Kaiso, and immune properties of the tumor microenvironment (Fig. 7). This analysis was conducted through a nearest-neighbor analysis of TMA tissues co-stained for pan-cytokeratin (tumor), CD8 (killer T-cells), CD68 (macrophages), and PD-L1 (immune checkpoint regulator) (Fig. 7a, b). The *x* and *y*-coordinates (Fig. 7b) of the resultant combinations of the tumor and immune phenotypes (Supplementary Figs. 9 and 10) were then mapped and the frequency distribution of distances between each cellular phenotype was profiled (Fig. 7c). Notably, nearest-neighbor profiling showed significant coordination between cytoplasmic Kaiso and LC3A/B, and the proximity of PD-L1-positive CD8 cells near tumor compared to insignificant association with nuclear Kaiso and Race (Fig. 7c). Similarly, elevated levels of both cytoplasmic Kaiso and LC3A/B were associated with increased proximity of PD-L1-positive CD68 cells near tumor compared to insignificant association with nuclear Kaiso and Race (Fig. 7c). In a similar fashion nearest-neighbor profiling of total CD8 cells in proximity to PD-L1-positive tumor showed a significant association between elevated cytoplasmic Kaiso, LC3A/B and Race compared to the insignificant association with nuclear Kaiso (Fig. 7c). Finally, proximity profiling of total CD68 cells near PD-L1-positive tumor cells similarly shows a significant association with elevated cytoplasmic Kaiso and LC3A/B compared to nuclear Kaiso and Race (Fig. 7c). Given the known immunosuppressive role of PD-L1 expression in both immune cells and tumor^59–61,68,69^, these findings reveal a strong association between LC3A/B and cytoplasmic Kaiso expression and the potential for an immune-suppressive tumor microenvironment. Notably, all associations between Kaiso and LC3A/B are specific to PDL1-positive cells as the trends described above are not significant when comparing total CD8 nor CD68 cells (Supplementary Fig. 11).

## Discussion

In this work, we utilized a novel application of computational digital analysis and a unique cohort of breast cancer patients to define new relationships between biomarkers based on their differential subcellular distribution to define strong prognostic markers of breast cancer survival. This “bedside-to-bench” approach not only reveals biomarkers correlated with overall breast cancer survival and an immune-suppressed tumor microenvironment but provides new functional and mechanistic insights into the cellular processes linked to the biomarkers. The functional linkage of cytoplasmic Kaiso to LC3A/B and the tumor microenvironment uncovers a new area of investigation into the role and mechanism of Kaiso in breast cancer progression. Kaiso could have multiple roles in promoting tumor progression through both modulations of transcription and autophagy-related events including (1) autophagy-mediated degradation pathways; and (2) secretory autophagy pathways dependent on LC3 conjugation. The precise mechanism of how Kaiso influences LC3 maturation will require further investigation. One possibility is that it may act as a scaffold to facilitate enzymatic lipid conjugation of LC3 proteins in the cytoplasm. Such a possibility is supported by the computational identification of a predicted LC3 interaction domain (LIR)^70^ in the C-terminal region of Kaiso adjacent to the DNA-binding zinc fingers. The possible dual role for Kaiso in autophagy and LC3-mediated secretion are not necessarily mutually exclusive since recent studies indicate that LDELS can occur independently of autophagy based on the observation that gene deletions that impair autophagy initiation do not block LDELS^44^. Other observations that will require further investigation arise from the morphological studies of cell lines and tumors. Notably, immunofluorescent staining for Kaiso reveals staining on tubulin (Fig. 4g and Supplementary Fig. 8) consistent with prior reports of the association of Kaiso with centrosomes^19^. Other observations include the accumulation of Kaiso at focal adhesion-like structures or assemblies reminiscent of actin-containing cell protrusion sites^18^ in MCF-7 (Supplementary Fig. 8). Such associations have not been previously described, but suggest a linkage between autophagy-related processes and the cycling of cell-matrix adhesion machinery^71^. Another interesting finding is the detection of race-specific differences in the tumor environment where there is a trend to a more suppressed immune microenvironment in women of African ancestry, particularly when considering CD8 cells near PD-L1-positive tumor (Fig. 7c). The differential contribution of race in the association of nuclear and cytoplasmic Kaiso with overall breast cancer survival (Fig. 6d, g) may contribute in part to this trend.

The general findings presented in this study, including the discovery of the prognostic significance of Kaiso subcellular partitioning and its linkage to immune-suppressive features of the tumor microenvironment, highlight its potential as a predictive biomarker to guide future treatment decisions, particularly in the use of immune checkpoint inhibitors. These results provide support for future applications in prospective studies where profiles of nuclear and cytoplasmic Kaiso are evaluated in clinical trials as both a predictive and prognostic breast cancer biomarker^72^. Furthermore, because the predictive value of nuclear and cytoplasmic Kaiso varies across racial groups, these findings further emphasize the need for the inclusion of diverse racial and ethnic groups in clinical trials.

## Methods

### Study population, tissue microarray construction, and analysis

Following IRB approval from East Carolina University and the National Institutes of Health intramural research program, de-identified formalin-fixed and paraffin-embedded (FFPE) tissue samples and de-identified clinical information abstracted from the medical records were requisitioned and initially procured for 733 breast cancer patients who underwent surgery for Stage 0 to Stage IV breast cancer between 2001 and 2010 at Pitt County Memorial Hospital (now Vidant Medical Center), Greenville, NC. Race, ethnicity, or “ancestry” was self-reported at the initial visit and captured in the medical record. Survival was recorded retrospectively from the medical records and the cancer registry. All patient samples and data obtained were de-identified and approved by the East Carolina University Institutional Review Board as a human subject exempt project, for which no informed consent is needed. The study was conducted in accordance with the Declaration of Helsinki. Race and/or ethnicity was self-reported at the initial visit and captured in the medical record. Survival was recorded retrospectively from the medical records and the cancer registry. The median follow-up is 8.5 years. 588 patient tumor blocks from this cohort were found suitable for use in the construction of a tissue microarray. Replicate tissue microarrays were constructed using 1 mm cores per previously described methods^73,74^, with a representation inclusive of 555 patients. Detailed methods for IHC, scoring, and the assignment of clinical variables are provided in the Supplementary Methods.

### Patient tumor RNA-seq analysis

RNA-seq analysis was performed on RNA extracted from FFPE tissue blocks (Total *N* = 126; EA *N* = 61; AA *N* = 65, Other = 1)^6^. Following a review of H&E stained slides, areas for tumors with >80% nuclei were circled, and 2.5 × 2–3 mm tissue cores were extracted from the corresponding regions of FFPE tissue blocks. Cores were shipped to the Beijing Genomics Institute (BGI) (Beijing, China) where RNA was extracted and sequenced (60M paired-end reads per sample) as previously described^75,76^. Detailed methods are provided in the data supplement.

### Immunofluorescence

Cells were grown on 22-mm-glass coverslips in 6-well plates to ~80% confluency before exposure to appropriate drugs or vehicular controls. After washing with PBS, cells were fixed with 4% formaldehyde in PBS at room temperature (RT) for 10 min. Cells were permeabilized with 0.1% Triton X-100 for 10 min, then incubated with blocking medium (PBS, 0.1% Tween-20, 10% normal goat serum) for 30 min in a humidified chamber at RT. Blocked samples were then stained with mouse anti-Kaiso (Abcam, ab12723; 1:1000) and rabbit anti-LC3A/B (Abcam, ab128025; 1:1000) in antibody diluent (PBS, 0.1% Tween-20, 1% BSA) for 1 h at RT in a humidified chamber. Anti-LC3A/B antibody recognizes both LC3A and LC3B proteins. After appropriate PBS washing, cells were stained with anti-mouse Alexa Fluor 488 (Invitrogen, A-11001; 1:2000) and anti-rabbit Alexa Fluor 594 (Invitrogen, A-11002; 1:1000) secondary antibodies in a humidity chamber in the dark for 1 h at RT. Coverslips were washed and then mounted on slides using ProLong Gold antifade reagent (Molecular Probes, P36934). MDA-MB-231 and MCF-7 used in this study were obtained from ATCC and identify was validated by STRS profiling. All cells were tested and found to be free of mycobacterial contamination.

### Colocalization analysis

*Z*-stack images for each channel were captured at ×100 magnification with Immersol 518F (Zeiss) oil immersion using a Zeiss Axiovert 200M fluorescent microscope running AxioVision software. Slices with the clearest resolution were selected for further analysis. The background was removed by subtracting the mean gray value for each channel in an area containing no cells. Colocalization analysis was performed using the ImageJ plug-in, “JaCoP”, according to the developer’s instructions. Negative controls were achieved by rotating one channel at least 90° and re-running the analysis. Positive controls were achieved by running colocalization analysis on two of the same channels. All graphs are plotted as the mean Pearson’s coefficient of at least three independent experiments with error bars representing the standard error. Each MCF-7 image contains ~15–20 cells, and MDA-MB-231 images contain ~5–10 cells per image.

### Multi-spectral fluorescent imaging and nearest-neighbor analysis

We used the Ultivue UltiMapper I/O PD-L1 assay to collect the qmIF data. This kit uses the following antigens: CD8, CD68, PD-L1, pan-cytokeratin (panCK), and DAPI (DNA marker). The raw image data is collected at 20×. The fluorescent dye intensities are normalized to 0–255. Image analysis was performed using a commercial software package (HALO, Indica Labs) at full magnification. The TMA spots were decomposed into individual analysis regions using the TMA module, with an invalidation threshold of about 80–90% empty space. A coordinate system was established for each spot with the origin being the bottom-left corner of the square TMA boundary. A unit coordinate is equivalent to one pixel or 0.5 microns. Watershed nuclear identification was performed on the DAPI channel with a nuclear contrast threshold of 0.5, and nuclear segmentation aggressiveness of 92%. Nuclei are required to be between 10 and 250 μm^2^ in size. A cytoplasmic region was grown from the nuclear boundary up to a radius of 4.2 μm. Cells were required to be <500 μm^2^. The average stain intensity within the cytoplasmic region was measured, and the positive-dye status for the antigen was defined as follows: CD8 (15) CD68 (8), panCK (10), and PD-L1 (13). Overall, we observed low backgrounds and strong signals. Phenotypes are defined using the coincidence/anti-coincidence logic of the positive-dye status. The logical combination for the main cell types are stromal (not panCK), T-cell (CD8), macrophage (CD68), and tumor (panCK and not CD8 and not CD68). These four cell types have 3 sub-phenotypes, inclusive and PD-L1^+^ (and PD-L1) or PD-L1- (and not PD-L1). The result of the phenotyping analysis is a text file for each tissue sample consisting of entries listing information about each cell location, including the manual phenotyping result and raw staining intensities using the defined coordinate system. The cell-point location was taken as the center of the rectangle which fully bounds the cell.

### Statistics and reproducibility

The nearest-neighbor algorithm was implemented as follows: for a given pair of phenotypes P_1_, P_2_, each composed of cells (detected with thresholds on their staining intensities) with two coordinates, *k* in [1,2], we compute, for a given cell *C*_*i*_ belonging to P_1_, the Euclidean distances to all cells belonging to P_2_, excluding those whose distance is less than 0.05 microns to prevent cell-overlap (Supplementary Figs. 9 and 10). We keep the minimum distance value among those, which we call the nearest-neighbor distance, and repeat this process for each cell in P_1_ to form a distribution of nearest-neighbor distances, *d*_*i*_. Measures of central tendencies for *d*_*i*_ were recorded as well as a histogram of frequencies of *d*_*i*_ using a bin width of 2 pixels up to 120 pixels. A counting error was assigned to each bin as being the square root of the number of entries. The normalization was chosen to be the total number of cells in the sample core, such that the integral of the histogram is equal to the density of the base phenotype being considered.

Population statistics or the average histogram shape were obtained by computing the mean value for each bin given a sub-population sample. The counting error was propagated and summed in quadrature with the standard error of the mean. The combined error is shown in the shaded band. To establish a test of statistical significance between two different histograms, we first define a test statistic as being the summed log-likelihood that each bin in the distribution has the same mean between two sub-populations. The natural log of the *p*-value, or “likelihood”, from a *t*-test between the individual bin values is taken. If the hypothesis sub-population mean is larger, this likelihood is defined to be positive, else it is negative. Schematically, large positive likelihoods represent significant upward fluctuations while large negative likelihoods represent significant downward fluctuations. These likelihoods are then summed across all bins. This forms the observed (hypothesis) statistic. The summed log-likelihood was then recomputed for 1k iterations using randomly assigned sub-populations, which have the same number of patients as the test sub-population. This forms the null distribution for the test statistic. The final *p*-value reported is the one-sided integral of the resultant null distribution from the observed value (Supplementary Fig. 10). This method overestimates the *p*-value since real differences in the sub-population can be double-counted when building the null distribution. However, this method treats bin-to-bin correlations correctly since it samples from real data.

Patient baseline characteristics and disease factors were summarized using descriptive statistics. Categorical variables were compared using the two-sided Pearson *χ*^2^ test. A comparison of IHC scoring was performed by a two-sided *t*-test and plotted as previously described^77^. Univariate and multivariate Cox proportional-hazards model was used to test the independent and combined prognostic values of proteins of interest with/without the presence of selected clinical variables. Spearman rank correlations were used to assess the relationship between protein *H*-score and gene expression (RPKM) values^78^. The significance of individual hazard ratios was estimated by Wald’s test. Optimal cut-off points for *H*-score were determined as previously described^6,79^ (Supplementary Fig. 3). The solid lines and histogram present data for samples with levels higher (red) or lower (blue); the dashed lines present data for samples divided into two groups (higher-red or lower-blue) based on the “optimal cut-off” algorithm^79^. Unsupervised hierarchical clustering of IHC protein score from all breast cancer samples was performed using complete linkage and distance correlations with the number of bootstrap replications (*n* = 1000) using the ‘pvclust’ R package^80^. The estimated clustering stability is measured by AU (approximately unbiased) (red) *p*-value and BP (bootstrap probability) (green) value for each cluster in a dendrogram^80^ (Supplementary Fig. 12). To explore the expression value together with clinical-pathological information, a heatmap was drawn where patients were arranged based on the order of the hierarchical clustering outcome.

### Gene set enrichment analysis of patient RNA-Seq data

The median cut-off of protein data was used to classify patients into two groups based on *H*-scores (e.g., low versus high Kaiso cytoplasmic) and mRNA abundance (RNA-seq). A two-sided *t*-test was performed, and all available genes were ranked according to *p*-value (lowest to highest). The *p*-value ranked gene list was used for functional correlation using the GSEA software (http://software.broadinstitute.org/gsea/index.jsp).

### Gene set enrichment analysis of MDA-MB-231 cell line gene expression

Four (*N* = 4) RNA samples each from WT MBA-MB-231 and RNAi Kaiso-depleted MBA-MD-231 cells were analyzed on the Affymetrix Human Gene 2.0 ST microarray and CEL files generated were normalized to produce gene-level expression values with the Robust Multiarray Average from the affy package to pre-process arrays and the limma package for identifying differentially expressed genes^81,82^. Relative fold depression of Kaiso (ZBTB33) mRNA was a 6-fold reduction. The hypergeometric test and Gene Set Enrichment Analysis (GSEA)^83^ was used to identify enriched signatures using the different pathway collections in the MSigDB database^84^. The GSEA pre-ranked method from GSEA was applied for this analysis. Human Gene 2.0 ST microarray of WT versus RNAi depleted MDA-MB-231 shows significant concordance with scrambled hairpin RNAi expressing MDA-MB-231 cells compared to Kaiso-depleted cell lines analyzed on the Nanostring DGE platform (Supplementary Fig. 6 and Supplementary Table 7).

### Genetic admixture analysis

For admixture analysis, RNA-Seq reads from 136 breast cancer patients were aligned to hg19 using STAR v2.5.2b^85^ with subsequent variant calling completed using GATK (v3.8) HaplotypeCaller^54,86^. After variant calling, Admixture v1.3.0^55^ was used to estimate ancestry proportions based on reference populations from the 1000 Genomes Project phase 3^87^ super populations. Rare variants (i.e., <5% across all phase 3 1000 genomes), all INDELs, and any SNPs that were not biallelic were removed before analysis.

### Reporting summary

Further information on research design is available in the Nature Research Reporting Summary linked to this article.

## Supplementary information

Supplementary Information

Description of Supplementary Files

Supplementary Data 1

Supplemental Data 2

Supplementary Data 3

Supplementary Data 4

Supplementary Data 5

Supplementary Data 6

Supplementary Data 7

Reporting Summary

## Data Availability

RNA-seq data are available at SRA archives https://www.ncbi.nlm.nih.gov/sraSRP158272. Proteomic and immunofluorescent intensity (*x*,*y*) coordinate point cloud data for nearest-neighbor analysis (Figs. 1–7) has been uploaded to figshare https://figshare.com/s/b5652eb7712fa83cf8bc. Additional supplemental data for Figs. 1–6 are also provided as Supplementary Data 1–7. Additional clinical source data and custom program code used to generate figures will be made available upon request (contact: SK Singhal).
