# Supplementary material for: Kaiso (ZBTB33) subcellular partitioning functionally links LC3A/B, the tumor microenvironment, and breast cancer survival
Source: Commun Biol. 2021 Feb 1;4:150. doi: 10.1038/s42003-021-01651-y (PMC7851134; doi:10.1038/s42003-021-01651-y)
Supplement: Supplementary file 1 — Supplementary Information [file 42003_2021_1651_MOESM1_ESM.pdf]

Supplementary Figures

Kaiso (*ZBTB33*) subcellular partitioning functionally links LC3A/B, the tumor microenvironment, and breast cancer survival

Sandeep K. Singhal[1,¶], Jung S. Byun[2,¶], Samson Park[2], Tingfen Yan[2‡], Ryan Yancey[3], Ambar Caban[3], Sara Gil Hernandez[2], Stephen M. Hewitt[4], Heike Boisvert[5], Stephanie Hennek[5], Mark Bobrow[5], Md Shakir Uddin Ahmed[6], Jason White[6], Clayton Yates[6], Andrew Aukerman[3], Rami Vanguri[3], Rohan Bareja[7], Romina Lenci[3], Paula Lucia Farre[8], Adriana De Siervi[8], Anna María Nápoles[2], Nasreen Vohra[9], and Kevin Gardner[3]*

[1]Department of Pathology, School of Medicine and Health Sciences, Department of Computer Science, School of Electrical Engineering and Computer Science, University of North Dakota, Grand Forks, ND 58202, USA. [2]Division of Intramural Research, National Institutes of Minority Health and Health Disparities, National Institutes of Health, Bethesda, MD 20892, USA. [3]Department of Pathology and Cell Biology, Columbia University Irvine Medical Center, New York, NY 10032, USA. [4]Laboratory of Pathology, Centers for Cancer Research, National Cancer Institute, National Institutes of Health, Bethesda, MD 20892, USA. [5]Ultivue, Inc. Cambridge, MA 02138, USA. [6]Department of Biology and Center for Cancer Research, Tuskegee University, Tuskegee, Al 36088, USA. [7]Department Computer Science Department, Columbia University, New York, NY 10032, USA. [8]Laboratorio de Oncologıa Molecular y Nuevos Blancos Terapeuticos, Instituto de Biologıa y Medicina Experimental (IBYME), CONICET, Argentina.[9]Brody School of Medicine, East Carolina University, Greenville, NC 27858, USA.
¶ These authors contributed equally

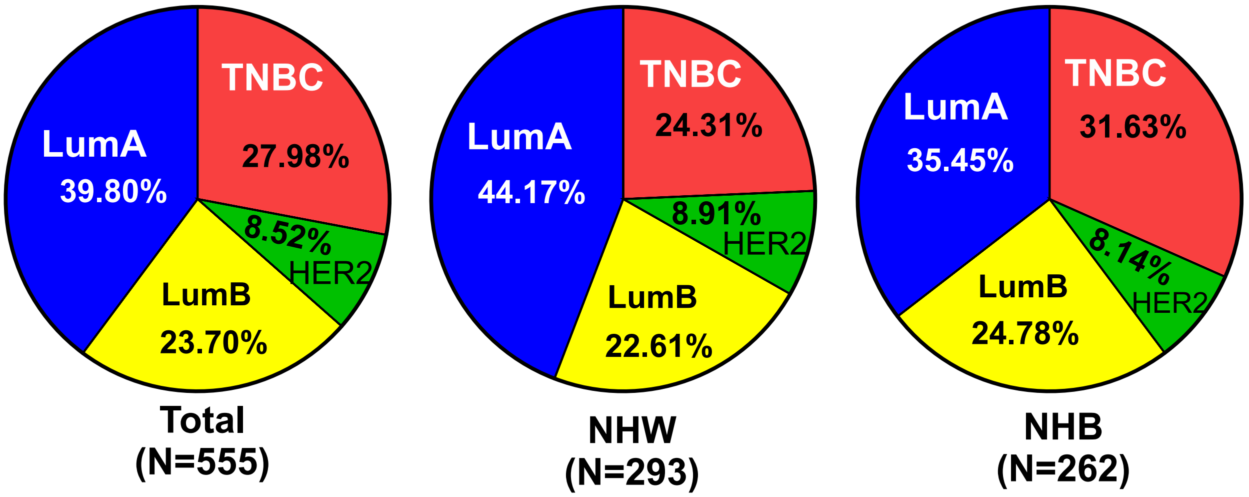

Supplementary Figure 1 Subtype and racial distribution of the breast cancer patient study cohort. LumA = Luminal A; LumB = Luminal B; TNBC = Triple negative breast cancer; HER2 = human EGF Receptor 2 positive tumor; NHW = non-hispanic White (self-reported); NHB = non-hispance Black (self-reported).

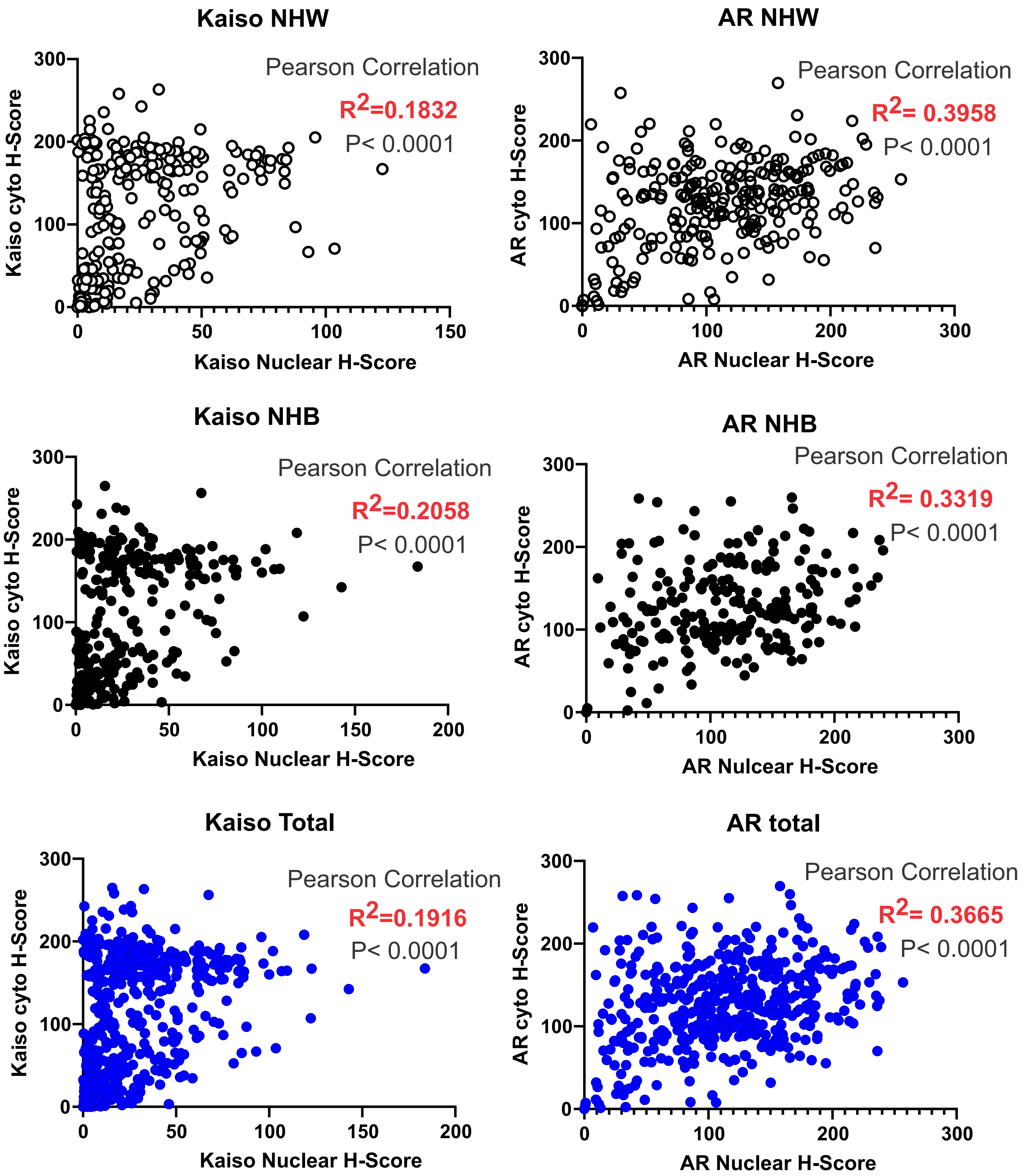

Supplementary Figure 2. Correlation (spearman) between nuclear and cytoplasmic Kaiso (left) and androgen receptors (AR) H-scores in non-hispanic white (NHW) and non-hispanic black (NHB), and total patient populations.

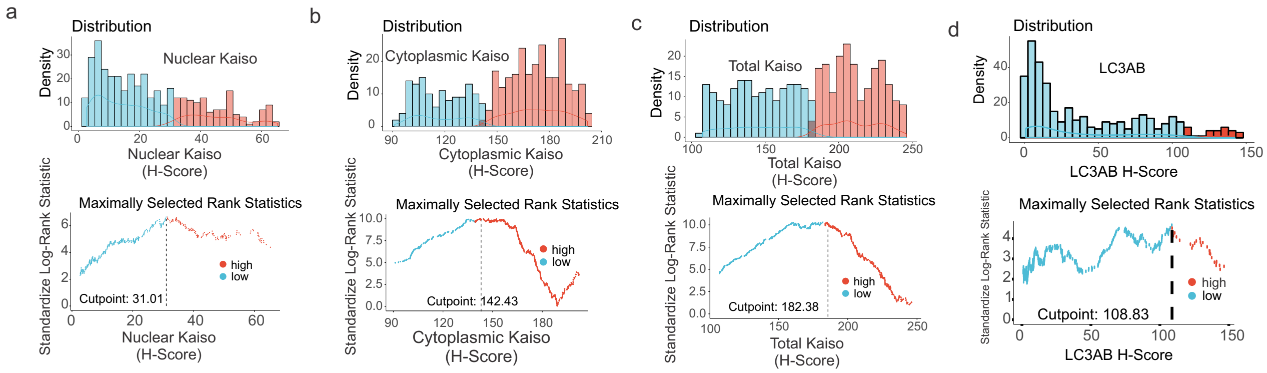

Supplementary Figure 3. Determination of maximally selected rank statistic to define optimal H-score cut-off for **a** nuclear Kaiso H-score; **b** Cytoplasmic Kaiso H-score; **c** Nuclear + Cytoplasmic H-score; and **d** LC3A/B H-Score.

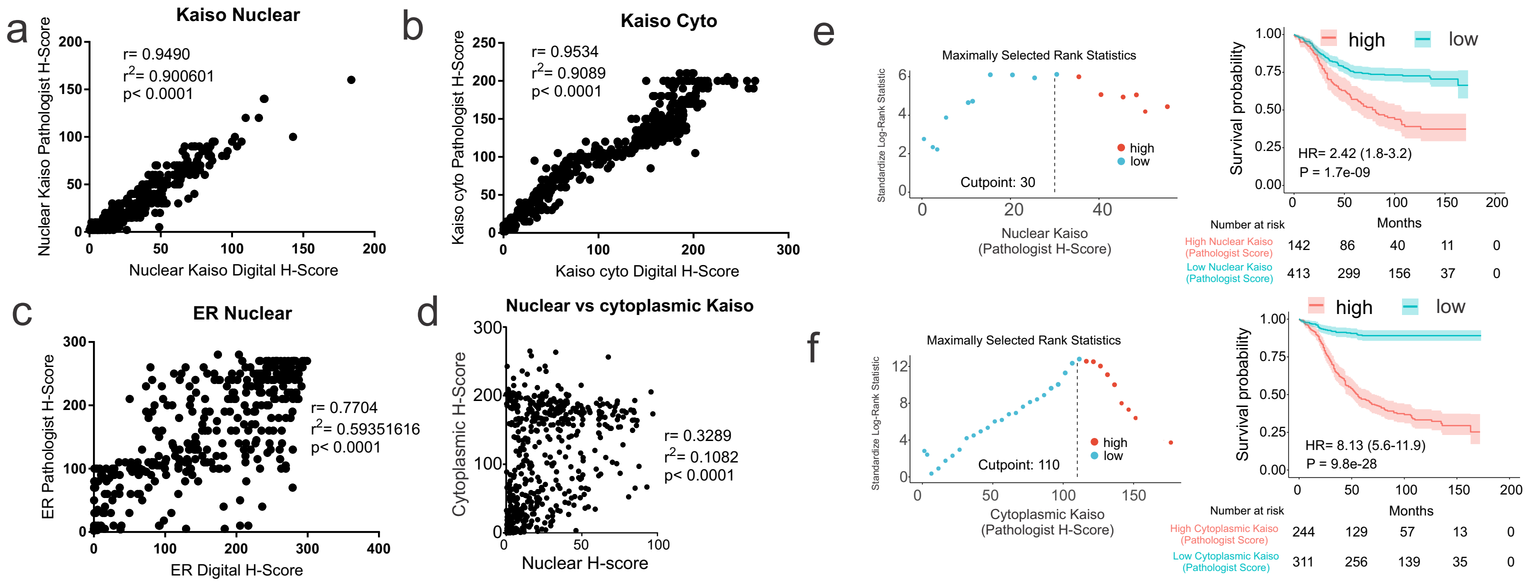

Supplementary Figure 4 Correlation of H-scores for nuclear Kaiso (**a**) and cytoplasmic Kaiso (**b**) between Pathologist's (y-axis) and digital (x-axis) scoring of IHC intensities generated by machine learning-based segmentation of pathologist annotated tumor regions into cytoplasmic and nuclear regions (Aperio TM) as previously described. Correlation of H-score for nuclear estrogen receptor (ER) and by Pathologist and digital IHC scoring (c). Correlation of cytoplasmic and nuclear Kaiso H-scores based on digital scoring.

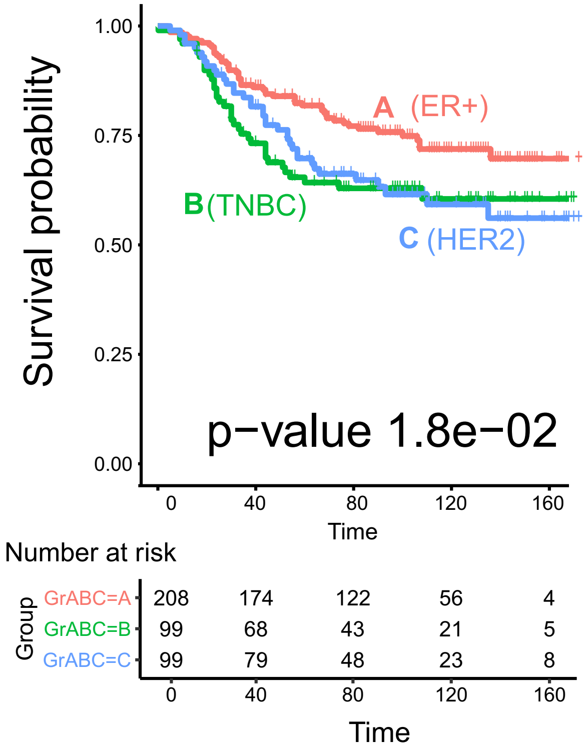

Supplementary Figure 5 Log-rank survival curve of major subgroup clusters identified by unsupervised clustering of antigen profiles in Figure 2a.

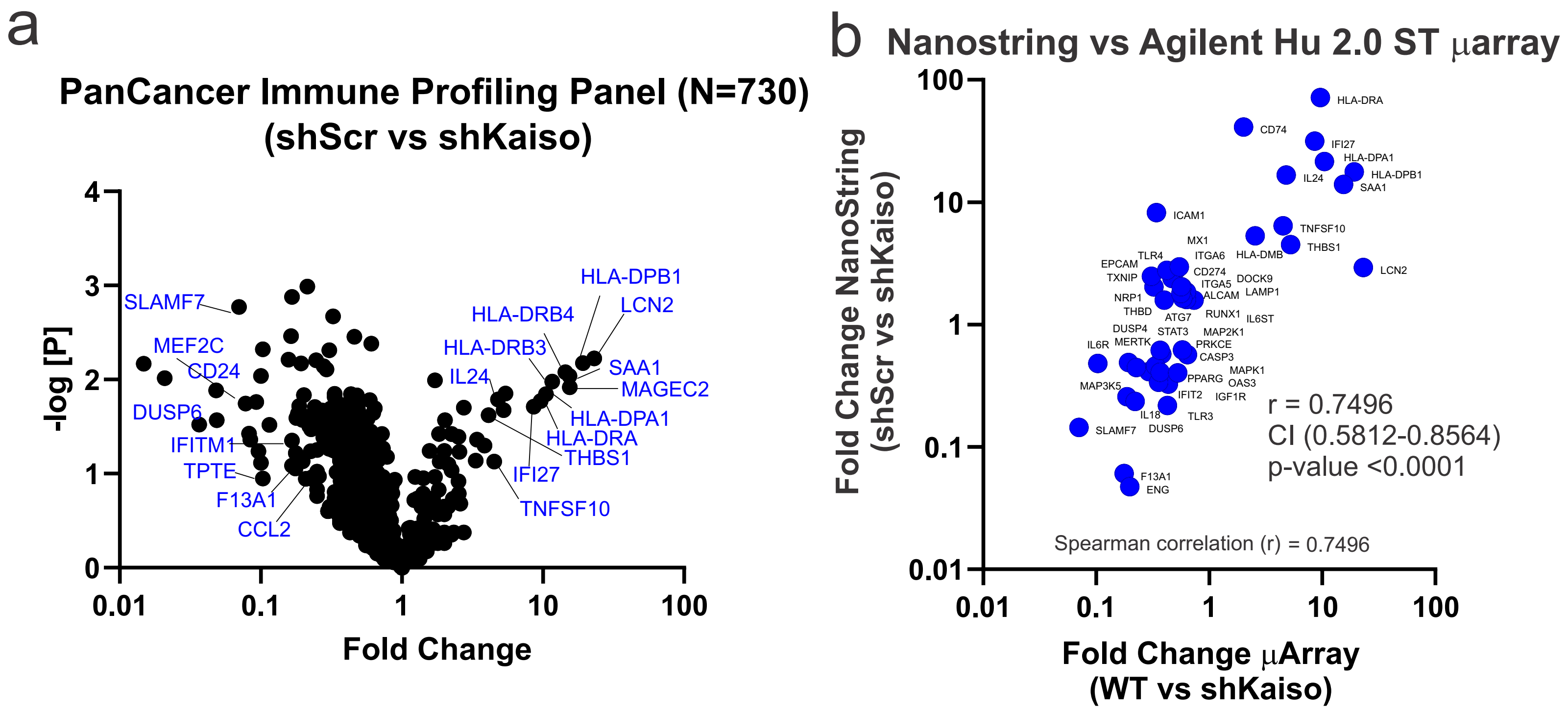

Supplementary Figure 6 (**a**) Differential expression profile of shKaiso MDA-MB-231 versus control MDA-MB-231 expressing scrambled non-targeting short hairpin (shScr) Genes highlighted in blue show genes in consensus with the differential expression profile of WT vs shKaiso in (Figure 3e). (**b**) Correlation between differential gene expression analysis between Nanostring PanCancer 730 gene Immune Profiling Panel and the Hu 2.0 ST µarray. Nonparametric correlation (Spearman= 0.7496) is shown

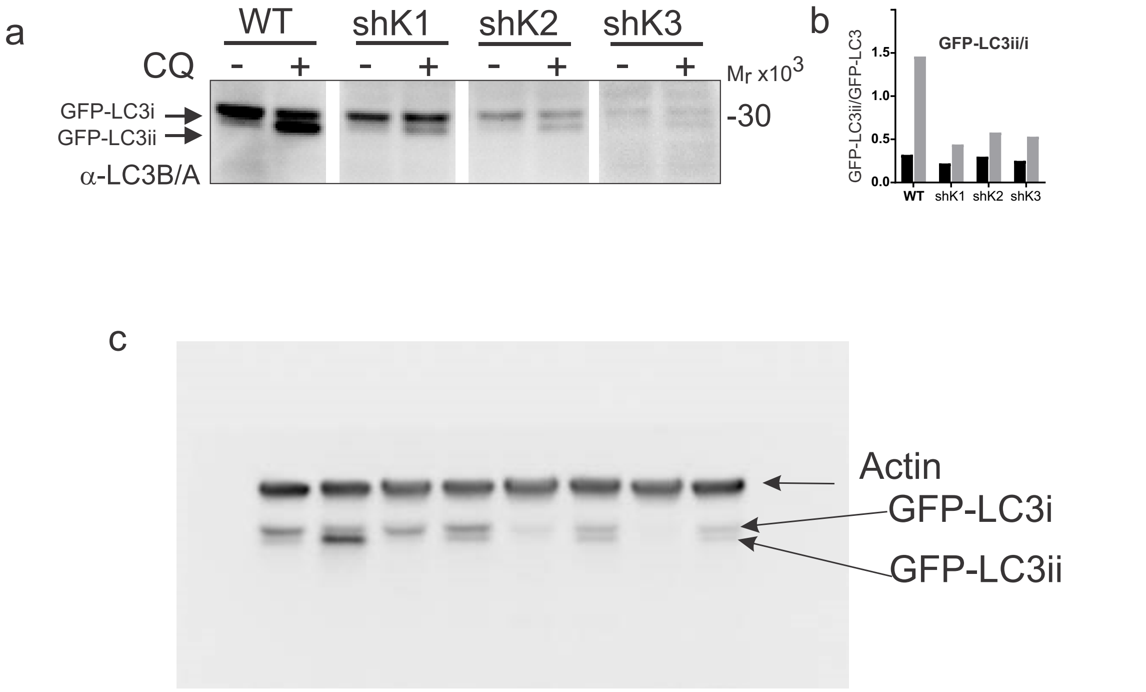

Supplementary Figure 7 **a** Immunoblot analysis of conversion of transfected GFP-LC3 from GFP-LC3i to GFP-LC3ii in WT MCF-7 compared to MCF-7 depleted of Kaiso by 3 different Kaiso short hairpins. **b** Densitometer profile of immunoblot. **c** source data for **Figure 4e**

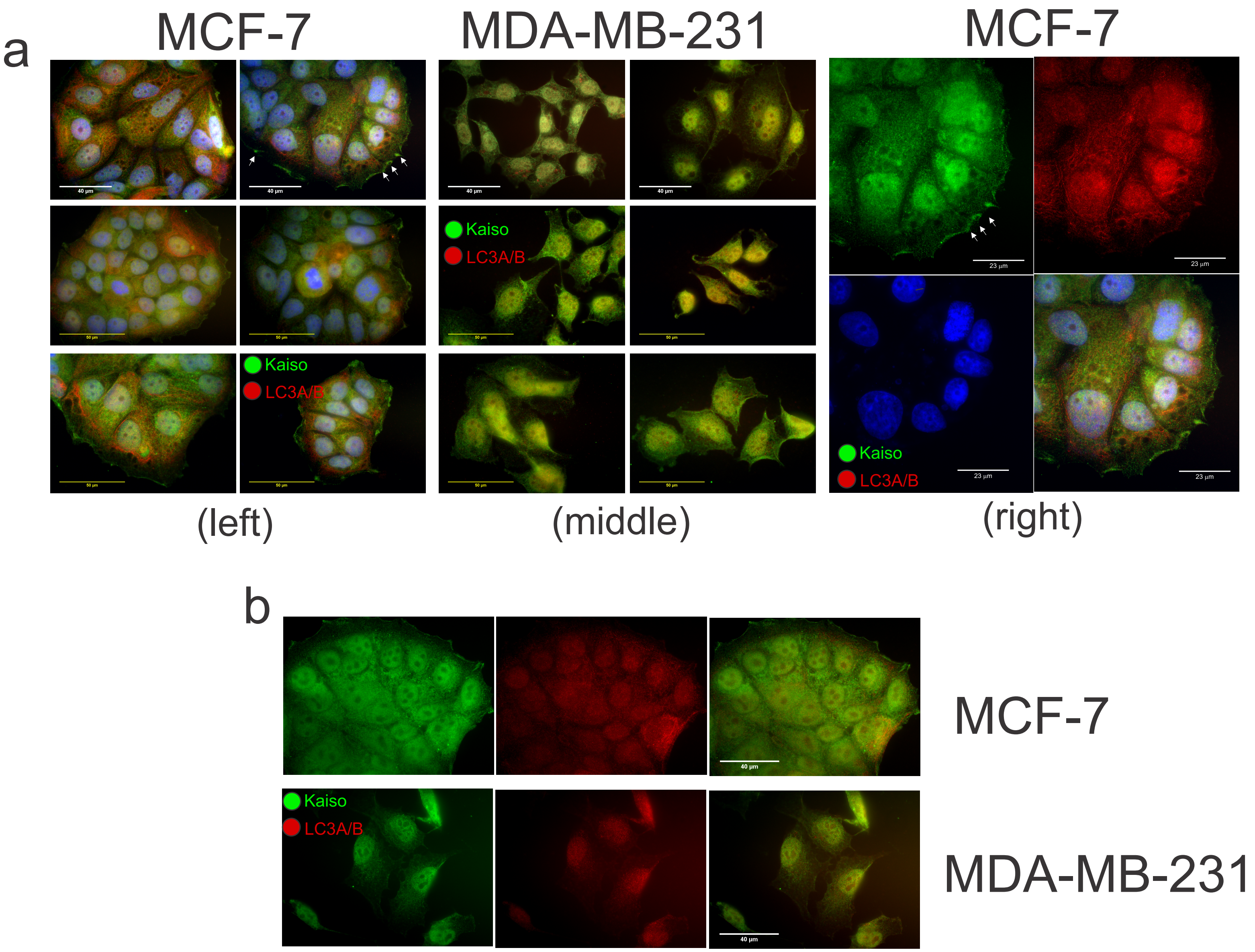

Supplementary Figure 8 (**a**) Additional fields of MCF-7 and (middle) MDA-MB-231 analyzed for co-localization and (right) and single channel immuno-fluorescence comparison of Kaiso (Green) and LC3A/B (Red) in MCF-7. (arrowhead indicate focal adhesion-like structures stained for Kaiso). (**b**) Kaiso (Green) and LC3A/B (Red) immuno-fluorescent images of MCF-7 (top) and MDA-MB-231 cells (bottom) treated in the absence of chloroquine.

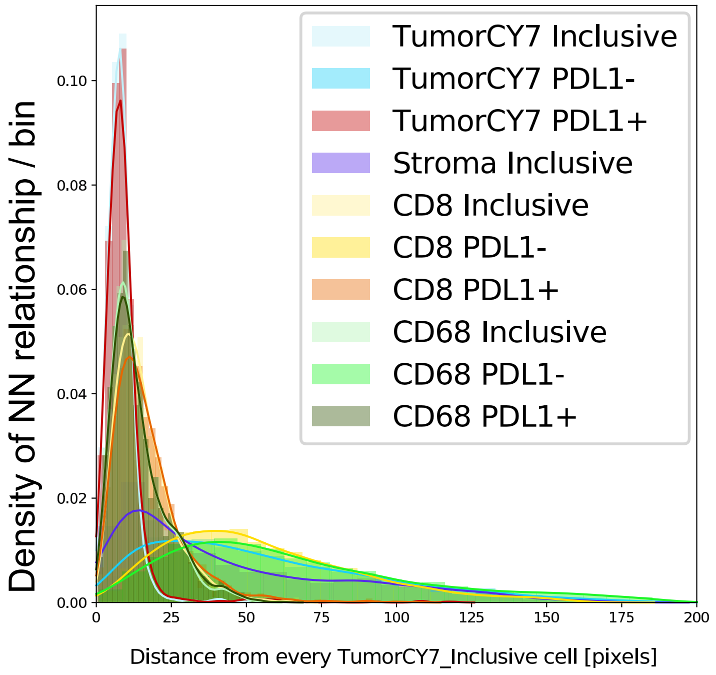

Supplementary Figure 9 The major immuno-phenotypes spatially profiled in the Nearest-Neighbor Analysis. TumorCy7 inclusive = total tumor; TumorCy7 PDL1- = PDL1 negative tumor; TumorCy7 PDL1+ = PDL1 positive Tumor; Stromal inclusive = all nuclei in the stroma; CD8 inclusive = total CD8 cells; CD8 PDL1- = PDL1 negative CD8 cells; CD8 PDL1+ = PDL1 positive CD8 cells; CD68 inclusive = total CD68 cells; CD68 PDL1- = PDL1 negative CD68 cells; CD68 PDL1+ = PDL1 positive CD68 cells.

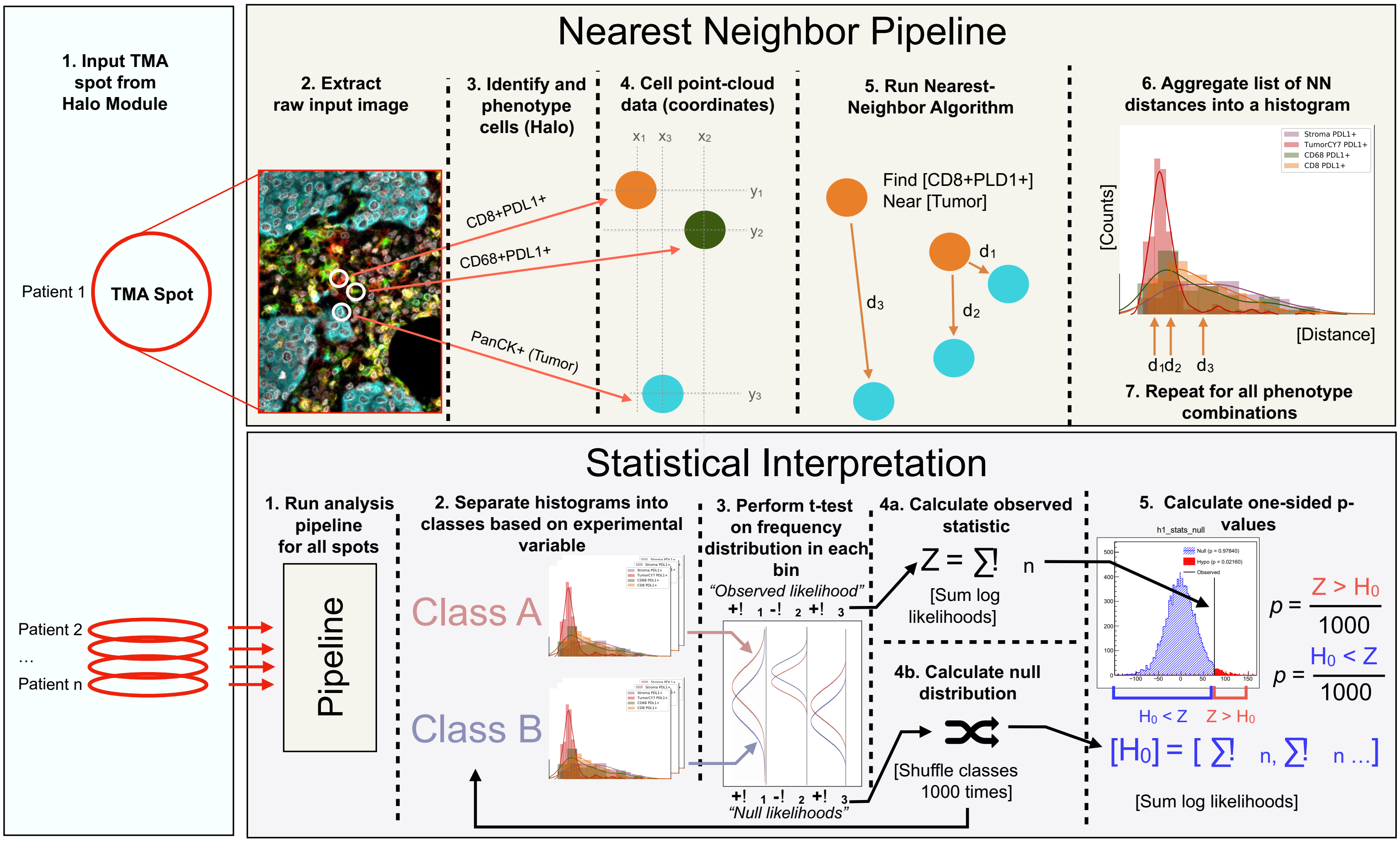

Supplementary Figure 10 Outline of the statistical analysis pipeline for Figure 7. See methods.

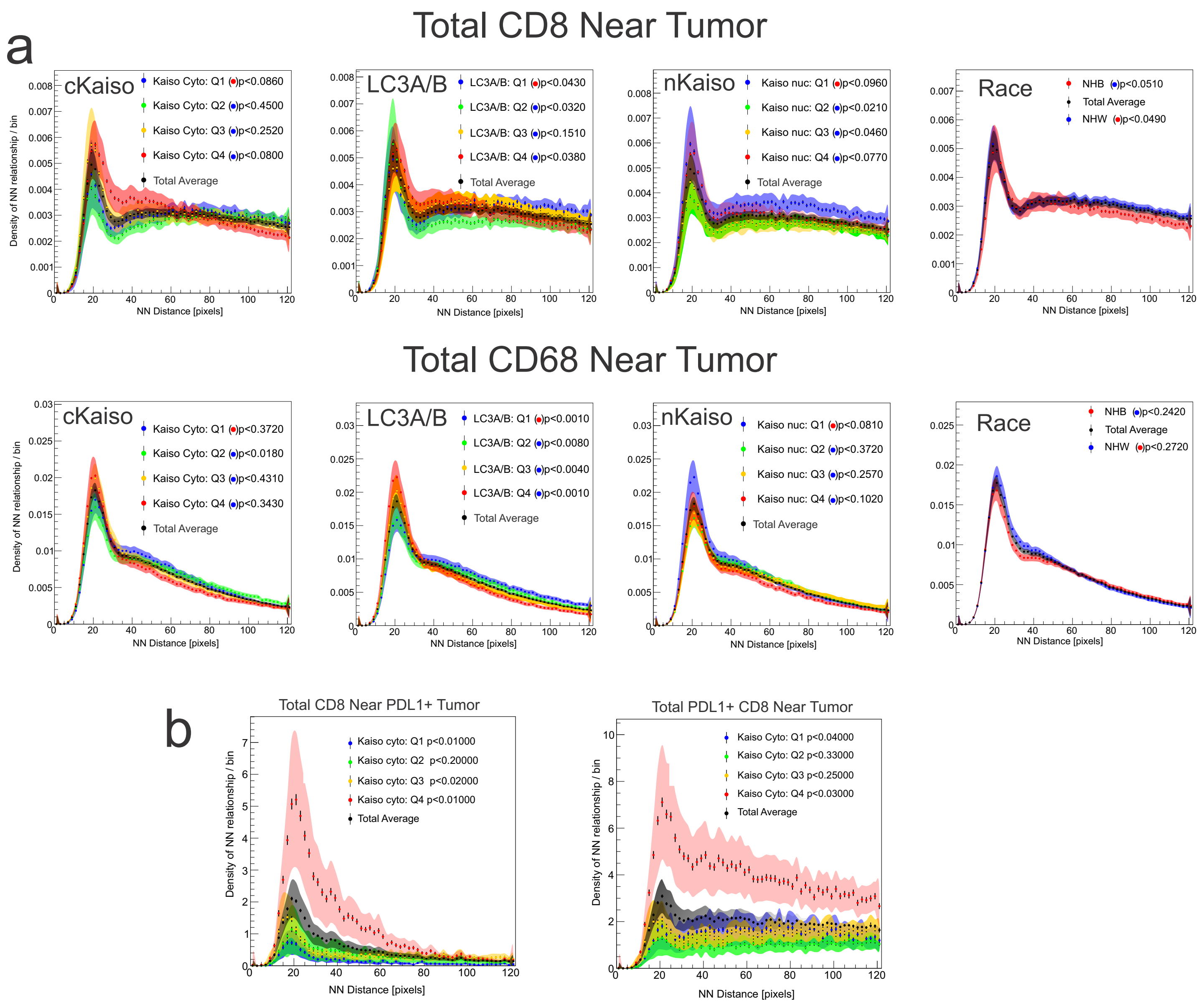

Supplementary Figure 11 (**a**) Nearest neighbor profiling of frequency distribution of the distance of total CD8 cells to total tumor (Top) or total CD68 cells to total tumor in breast cancer patients stratified by Nuclear Kaiso, Cytoplasmic Kaiso, LC3A/B staining and Race. (**a**)

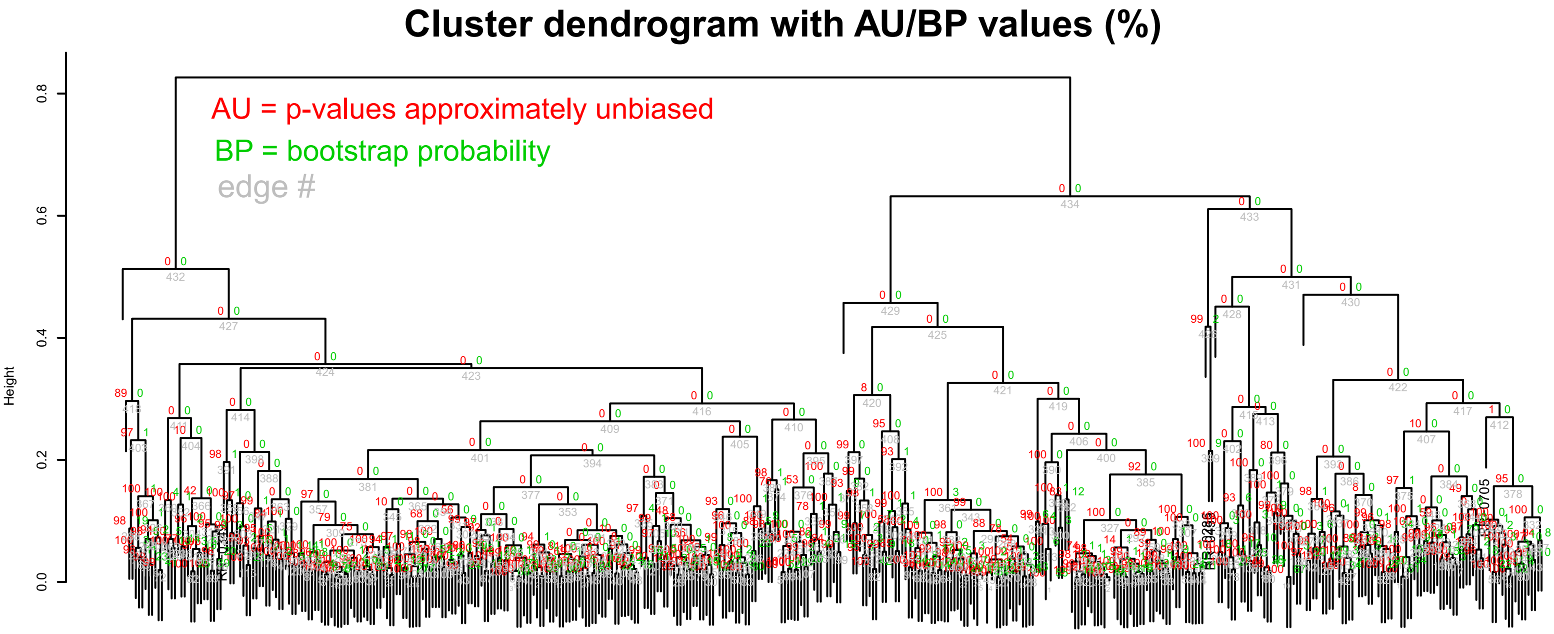

Supplementary Figure 12 Stability of unsupervised hierarchical clustering of IHC protein score from all breast cancer samples (**Figure 2)** performed using complete linkage and distance correlations with the number of bootstrap replications (n=1000). The estimated the clustering stability is measured by AU (approximately unbiased) (red) p-value and BP (bootstrap probability) (green) value for each cluster in a dendrogram

Supplementary Methods

Kaiso (*ZBTB33*) subcellular partitioning functionally links LC3A/B, the tumor microenvironment, and breast cancer survival

Sandeep K. Singhal[1,15], Jung S. Byun[2,15], Samson Park[2], Tingfen Yan[2ǂ], Ryan Yancey[3], Ambar Caban[3], Sara Gil Hernandez[2], Stephen M. Hewitt[4], Heike Boisvert[5], Stephanie Hennek[5], Mark Bobrow[5], Md Shakir Uddin Ahmed[6], Jason White[6], Clayton Yates[6], Andrew Aukerman[3], Rami Vanguri[3], Rohan Bareja[7], Romina Lenci[3], Paula Lucia Farre[8], Adriana De Siervi[8], Anna María Nápoles[2], Nasreen Vohra[9], and Kevin Gardner[3]*

Tissue Microarray Construction

Following review of hematoxylin and eosin stained sections, regions of interest were outlined, and 1 mm cores were removed from corresponding blocks using a Pathology Devices TMArrayer (Westminster, MD). Replicate TMA blocks were constructed. All arrays contained appropriately chosen positive and negative control tissue. Digital image analysis and scoring of IHC staining was performed using Leica Aperio digital analysis platforms, in which 3 representative regions of tumor were outlined on each core by a pathologist and scored digitally using the Nuclear v9 algorithm to generate a histo-score (H-Score; 0-300) based on the percent of positive cells with assigned intensity thresholds of negative (0), low (1), moderate (2) or high (3) as previously described[1]. Staining intensities across TMA slide sets were adjusted to control tissue present on each slide. To estimate the degree of scoring variation due to tumor heterogeneity, we performed repeat measurements of separate tumor cores from 1/3 of the patients. Analysis of this data by the Root Mean Square Error (RMSE) method indicates a scoring variation less than 24.15% for antigens scored.

Methods for Clinical Variables

Median follow-up and median survival for patients was 8.5 and 6.67 years respectively. Clinical subtypes[2,3] were categorized as HER2+ when available information in the patient medical record

showed a score greater than 2 by in situ hybridization or 3+ by immunohistochemistry. ER+ patients were classified from the medical record and confirmed by IHC: missing data was replaced by TMA All digital scoring was performed using the nuclear and membrane algorithms provided by the Leica Biosystems Aperio software. Areas of interest were outlined by the pathologist and then scored independently by the pathologist and the respective algorithms.

Methods for Immunohistochemistry

Breast tumor tissue microarrays were stained, using monoclonal primary antibodies, ERα at 1:35, low pH (Catalog No. MA5- 13191, DAKO), EGFR at 1:500, high pH (Catalog No. M7298, DAKO), E-Cadherin at 1:50, high pH (Catalog No. M361201-2, DAKO) and GATA3 at 1:50, high pH (Catalog No. sc-268, Santa Cruz Biotechnology). Also, polyclonal primary antibodies were used, FOXA1 at 1:10,000, high pH (Catalog No. ab23738, Abcam) and the ready-to-use antibody c-erbB-Oncoprotein at a high pH (Catalog No. A0485, DAKO). Kaiso (6F / 6F8, Abcam ab12723) antibody staining for IHC was performed at 1:1000 dilution at high pH. Anti-LC3A/B antibody (LC3A/B (D3U4C) XP® Rabbit mAb, Cell Signaling Technology) was used at 1:300 dilution at high pH.

RNA-seq

Following review of H&E stained slides areas of tumor with >80% nuclei were circled, and 2.5 x 2-3 mm tissue cores were extracted from the corresponding regions of FFPE tissue blocks. Sample cores were shipped to BGI Beijing Genome Institute (BGI) for further processing as previously described[4,5]. Briefly, the total RNA samples were first treated with DNase I, followed by a mRNA enrichment step enriched by using the oligo (dT) coupled magnetic beads. Following fragmentation, the first strand of cDNA was synthesized by using random hexamer-primers. Buffer, dNTPs, RNase H and DNA polymerase I were added to synthesize the second strand. The double strand cDNA was

purified with magnetic beads and end reparation and 3'-end single nucleotide A (adenine) addition was then performed. Finally, sequencing adaptors were ligated to the fragments. The fragments were enriched by PCR amplification. During the QC step, Agilent 2100 Bioanalyzer and ABI StepOnePlus Real-Time PCR System were used to qualify and quantify the sample library. The library was sequenced (60 M paired-end read per sample) on an Illumina HiSeqTM4000.

Sequence Data Analysis

After sequencing, the raw reads were filtered (BGI). Data filtering included removing adapter sequences, contamination and low-quality reads from raw reads. The cleaned reads (fastQ) were mapped to reference sequence using HISAT[6]. Raw reads and RPKM for each sample were calculated using HOMER[7]. Differential gene expression was performed using EdgeR Bioconductor [8]. Sequences were submitted to BioProject Dbase under BioProject ID: PRJNA486351 and submissionID: SUB4408142 for public availability.

Statistical Analysis

A Spearman rank correlation test was performed to test the relation between its protein H-score and gene expression (RPKM value) values[9]. A completely unsupervised hierarchical clustering approach was performed on the 486 patient sample H-scores containing complete clnical information. Complete linkage and distance correlations were used for clustering protein data with bootstrap resampling techniques. The stability of the clustering was estimated with the 'pvclust' R package[10] available on CRAN (https://cran.r-project.org/web/packages/pvclust/pvclust.pdf). A two-sided t-test was employed to test the null hypothesis (H_0) assumption of equality of the protein values in two defined groups of data and demonstrated by violin plots using R software and ggplot2 package[11].

To classify the patients into low versus high-risk categories using selected protein H-scores, the optimal cutoff approach[12] has been used to compute optimal cutoff-points for diagnostic markers with

continuous values for the entire population. The same cutoff-points were applied to subclasses of data i.e. the NHB and NHW populations. In addition, we performed a prognostic value comparative analysis using optimum cutoff-point based on a specific population as well as the median of the entire population. The prognostic value of proteins or genes were calculated by univariate Cox regression. A multivariate Cox proportional-hazards model[13] was used to test the independent and combined prognostic values of proteins of interest with/without the presence of selected clinical variables. Cox models were stratified by race to account for the possible heterogeneity in patient selection or other potential confounders. The 'survival' R package was used which is available on CRAN (https://cran.r-project.org/web/packages/survival/survival.pdf). The significance of individual hazard ratios was estimated by Wald's test.

RNA interfence

Sequences used for RNAi studies to deplete Kaiso (*ZBTB33*) are as follows: shKaiso1 5'-TTGACTATGAACTGACTTT-3';shKaiso2 5'-AACACTGATACCTTCGCTC-3'; shKaiso3 5'-TATAGTAGCCCCATTATCT-3'.

Supplementary References

1. McCarty KS, Jr., Miller LS, Cox EB, et al: Estrogen receptor analyses. Correlation of biochemical and immunohistochemical methods using monoclonal antireceptor antibodies. Arch Pathol Lab Med 109:716-21, 1985
2. Goldhirsch A, Winer EP, Coates AS, et al: Personalizing the treatment of women with early breast cancer: highlights of the St Gallen International Expert Consensus on the Primary Therapy of Early Breast Cancer 2013. Ann Oncol 24:2206-23, 2013
3. Goldhirsch A, Wood WC, Coates AS, et al: Strategies for subtypes--dealing with the diversity of breast cancer: highlights of the St. Gallen International Expert Consensus on the Primary Therapy of Early Breast Cancer 2011. Ann Oncol 22:1736-47, 2011
4. Jia W, Qiu K, He M, et al: SOAPfuse: an algorithm for identifying fusion transcripts from paired-end RNA-Seq data. Genome Biology 14:R12, 2013
5. Peng Z, Cheng Y, Tan BC-M, et al: Comprehensive analysis of RNA-Seq data reveals extensive RNA editing in a human transcriptome. Nature Biotechnology 30:253, 2012
6. Kim D, Langmead B, Salzberg SL: HISAT: a fast spliced aligner with low memory requirements. Nat Methods 12:357-60, 2015
7. Heinz S, Benner C, Spann N, et al: Simple combinations of lineage-determining transcription factors prime cis-regulatory elements required for macrophage and B cell identities. Mol Cell 38:576-89, 2010
8. Robinson MD, McCarthy DJ, Smyth GK: edgeR: a Bioconductor package for differential expression analysis of digital gene expression data. Bioinformatics 26:139-40, 2010
9. Myers JL, Well A, Lorch RF: Research design and statistical analysis (ed 3rd). New York, Routledge, 2010

10. Suzuki R, Shimodaira H: Pvclust: an R package for assessing the uncertainty in hierarchical clustering. Bioinformatics 22:1540-2, 2006

11. Hintze JL, Nelson RD: Violin plots: A box plot-density trace synergism. American Statistician 52:181-184, 1998

12. Hothorn T, Lausen B: On the exact distribution of maximally selected rank statistics. Computational Statistics & Data Analysis 43:121-137, 2003

13. Cox DR: Regression Models and Life-Tables. Journal of the Royal Statistical Society Series B-Statistical Methodology 34:187-+, 1972
