# Supplementary material for: Kaiso (ZBTB33) subcellular partitioning functionally links LC3A/B, the tumor microenvironment, and breast cancer survival
Source: Commun Biol. 2021 Feb 1;4:150. doi: 10.1038/s42003-021-01651-y (PMC7851134; doi:10.1038/s42003-021-01651-y)
Supplement: Supplementary file 2 — Description of Supplementary Files [file 42003_2021_1651_MOESM2_ESM.pdf]

Description of Additional Supplementary Files

File name: Supplementary Data 1

Description: Kaiso LC3A/B RNA vs Protein

File name: Supplementary Data 2

Description: Log Rank Survival analysis data from Figure 2

File name: Supplementary Data 3

Description: Autophagy Gene list

File name: Supplementary Data 4

Description: Gene Ontology (GO) Gene Set Enrichment Analysis (GSEA) Data from MDA-MB-231 WT vs shKaiso RNA analysis

File name: Supplementary Data 5

Description: Gene Ontology (GO) Gene Set Enrichment Analysis (GSEA) Data from Patients stratified by Kaiso cytoplasmic protein

File name: Supplementary Data 6

Description: Gene Ontology (GO) Gene Set Enrichment Analysis (GSEA) Data from Patients stratified by Kaiso nuclear protein

File name: Supplementary Data 7

Description: Nanostring DGE immune SCR vs ShKaiso
