# Supplementary material for: Kaiso (ZBTB33) subcellular partitioning functionally links LC3A/B, the tumor microenvironment, and breast cancer survival
Source: Commun Biol. 2021 Feb 1;4:150. doi: 10.1038/s42003-021-01651-y (PMC7851134; doi:10.1038/s42003-021-01651-y)
Supplement: Supplementary file 4 — Supplemental Data 2 [file 42003_2021_1651_MOESM4_ESM.docx]

| Paired |  |  |  |  |  |  |  |  |  |
| --- | --- | --- | --- | --- | --- | --- | --- | --- | --- |
|  | coef | exp(coef) | exp(-coef) | lower .95 | upper .95 | se(coef) | z | Pr(>\|z\|) |  |
| GroupA2 | 0.7434 | 2.103 | 0.4755 | 0.897 | 4.931 | 0.4348 | 1.71 | 0.08727 | . |
| GroupB1 | 1.2956 | 3.653 | 0.2737 | 1.4134 | 9.443 | 0.4845 | 2.674 | 0.00749 | ** |
| GroupB2 | 1.3212 | 3.748 | 0.2668 | 1.4747 | 9.526 | 0.4759 | 2.776 | 0.0055 | ** |
| GroupB3 | 0.4183 | 1.519 | 0.6582 | 0.4279 | 5.394 | 0.6465 | 0.647 | 0.51763 |  |
| GroupC1 | 0.3639 | 1.439 | 0.6949 | 0.4385 | 4.722 | 0.6063 | 0.6 | 0.54834 |  |
| GroupC2 | 1.2987 | 3.664 | 0.2729 | 1.529 | 8.782 | 0.4459 | 2.912 | 0.00359 | ** |
| Overall |  |  |  |  |  |  |  |  |  |
| Likelihood ratio test= 19.72 on 6 df, p=0.003 | | | |  |  |  |  |  |  |
| Wald test = 18.06 on 6 df, p=0.006 | | | |  |  |  |  |  |  |
| Score (logrank) test = 19.35 on 6 df, p=0.004 | | | |  |  |  |  |  |  |
| Wald test = 18.06 on 6 df, p=0.006 | | | |  |  |  |  |  |  |
| Score (logrank) test = 19.35 on 6 df, p=0.004 | | | |  |  |  |  |  |  |
