# Supplementary material for: Kaiso (ZBTB33) subcellular partitioning functionally links LC3A/B, the tumor microenvironment, and breast cancer survival
Source: Commun Biol. 2021 Feb 1;4:150. doi: 10.1038/s42003-021-01651-y (PMC7851134; doi:10.1038/s42003-021-01651-y)
Supplement: Supplementary file 10 — Reporting Summary [file 42003_2021_1651_MOESM10_ESM.pdf]

Corresponding author(s): Kevin Gardner, M.D.,Ph.D.

Last updated by author(s): 12/07/2020

Reporting Summary

Nature Research wishes to improve the reproducibility of the work that we publish. This form provides structure for consistency and transparency in reporting. For further information on Nature Research policies, see Authors & Referees and the Editorial Policy Checklist .

Statistics

For all statistical analyses, confirm that the following items are present in the figure legend, table legend, main text, or Methods section.

n/a	Confirmed	
☐	☒	The exact sample size (*n*) for each experimental group/condition, given as a discrete number and unit of measurement
☐	☒	A statement on whether measurements were taken from distinct samples or whether the same sample was measured repeatedly
☐	☒	The statistical test(s) used AND whether they are one- or two-sided<br>*Only common tests should be described solely by name; describe more complex techniques in the Methods section.*
☐	☒	A description of all covariates tested
☐	☒	A description of any assumptions or corrections, such as tests of normality and adjustment for multiple comparisons
☐	☒	A full description of the statistical parameters including central tendency (e.g. means) or other basic estimates (e.g. regression coefficient) AND variation (e.g. standard deviation) or associated estimates of uncertainty (e.g. confidence intervals)
☐	☒	For null hypothesis testing, the test statistic (e.g. *F*, *t*, *r*) with confidence intervals, effect sizes, degrees of freedom and *P* value noted<br>*Give P values as exact values whenever suitable.*
☐	☒	For Bayesian analysis, information on the choice of priors and Markov chain Monte Carlo settings
☐	☒	For hierarchical and complex designs, identification of the appropriate level for tests and full reporting of outcomes
☐	☒	Estimates of effect sizes (e.g. Cohen's *d*, Pearson's *r*), indicating how they were calculated

Our web collection on statistics for biologists contains articles on many of the points above.

Software and code

Policy information about availability of computer code

Data collection	Following IRB approval from East Carolina University and the National Institutes of Health intramural research program, de-identified formalin-fixed and paraffin-embedded (FFPE) tissue samples and de-identified clinical information abstracted from the medical records were requisitioned and initially procured for 733 breast cancer patients who underwent surgery for Stage 0 to Stage IV breast cancer between 2001 and 2010 at Pitt County Memorial Hospital (now Vidant Medical Center), Greenville, NC. Race, ethnicity or “ancestry” was self-reported at the initial visit and captured in the medical record. Survival was recorded retrospectively from the medical records and the cancer registry.
Data analysis	Colocalization Analysis. Z-stack images for each channel were captured at 100X magnification with Immersol 518F (Zeiss) oil immersion using a Zeiss Axiovert 200M fluorescent microscope running AxioVision software. Slices with the clearest resolution were selected for further analysis. The background was removed by subtracting the mean grey value for each channel in an area containing no cells. Colocalization analysis was performed using the ImageJ plug-in, “JaCoP”, according to the developer’s instructions. Negative controls were achieved by rotating one channel at least 90° and re-running the analysis. Positive controls were achieved by running colocalization analysis on two of the same channels. All graphs are plotted as the mean Pearson’s coefficient of at least three independent experiments with error bars representing the standard error. Each MCF7 image contains approximately 15 to 20 cells, and MDA-MB-231 images contain approximately 5 to 10 cells per image.<br><br>Sequence Data Analysis<br>After sequencing, the raw reads were filtered (BGI). Data filtering included removing adapter sequences, contamination and low-quality reads from raw reads. The cleaned reads (fastQ) were mapped to reference sequence using HISAT6. Raw reads and RPKM for each sample were calculated using HOMER7. Differential gene expression was performed using EdgeR Bioconductor 8. Sequences were submitted to BioProject Dbase under BioProject ID: PRJNA486351 and submissionID: SUB4408142 for public availability.<br>Statistical Analysis<br>A Spearman rank correlation test was performed to test the relation between its protein H-score and gene expression (RPKM value) values9. A completely unsupervised hierarchical clustering approach was performed on the 486 patient sample H-scores containing

complete clnical information. Complete linkage and distance correlations were used for clustering protein data with bootstrap resampling techniques. The stability of the clustering was estimated with the ‘pvclust’ R package10 available on CRAN (https://cran.r-project.org/web/packages/pvclust/pvclust.pdf). A two-sided t-test was employed to test the null hypothesis (H0) assumption of equality of the protein values in two defined groups of data and demonstrated by violin plots using R software and ggplot2 package11.

To classify the patients into low versus high-risk categories using selected protein H-scores, the optimal cutoff approach12 has been used to compute optimal cutoff-points for diagnostic markers with continuous values for the entire population. The same cutoff-points were applied to subclasses of data i.e. the NHB and NHW populations. In addition, we performed a prognostic value comparative analysis using optimum cutoff-point based on a specific population as well as the median of the entire population. The prognostic value of proteins or genes were calculated by univariate Cox regression. A multivariate Cox proportional-hazards model13 was used to test the independent and combined prognostic values of proteins of interest with/without the presence of selected clinical variables. Cox models were stratified by race to account for the possible heterogeneity in patient selection or other potential confounders. The ‘survival’ R package was used which is available on CRAN (https://cran.r-project.org/web/packages/survival/survival.pdf). The significance of individual hazard ratios was estimated by Wald’s test.

Multi-spectral fluorescent imaging and Nearest-Neighbor Analysis We used the Ultivue UltiMapper I/O PD-L1 assay to collect the qmIF data. This kit uses the following antigens: CD8, CD68, PD-L1, pan-cytokeratin (panCK), and DAPI (DNA marker). The raw image data is collected at 20x. The fluorescent dye intensities are normalized to 0-255. Image analysis was performed using a commercial software package (HALO, Indica Labs) at full magnification. The TMA spots were decomposed into individual analysis regions using the TMA module, with an invalidation threshold of about 80-90% empty space. A coordinate system was established for each spot with the origin being the bottom-left corner of the square TMA boundary. A unit coordinate is equivalent to one pixel or 0.5 microns. Watershed nuclear identification was performed on the DAPI channel with a nuclear contrast threshold of 0.5, and nuclear segmentation aggressiveness of 92%. Nuclei are required to be between 10 and 250 um2 in size. A cytoplasmic region was grown from the nuclear boundary up to a radius of 4.2 microns. Cells were required to be less than 500 um2. The average stain intensity within the cytoplasmic region was measured, and the positive-dye status for the antigen was defined as follows: CD8 (15) CD68 (8), panCK (10), and PD-L1 (13). Overall, we observed low backgrounds and strong signals. Phenotypes are defined using coincidence/anti-coincidence logic of the positive-dye status. The logical combination for the main cell types are stromal (not panCK), T-cell (CD8), macrophage (CD68), and tumor (panCK and not CD8 and not CD68). These four cell types have 3 sub-phenotypes, inclusive and PD-L1+ (and PD-L1) or PD-L1- (and not PD-L1). The result of the phenotyping analysis is a text file for each tissue sample consisting of entries listing information about each cell location, including the manual phenotyping result and raw staining intensities using the defined coordinate system. The cell-point location was taken as the center of the rectangle which fully bounds the cell.

Statistics and Reproducibility The nearest-neighbor algorithm was implemented as follows: for a given pair of phenotypes P1, P2, each composed of cells (detected with thresholds on their staining intensities) with two coordinates, k in [1,2], we compute, for a given cell Ci belonging to P1, the Euclidean distances to all cells belonging to P2, excluding those whose distance is less than 0.05 microns to prevent cell-overlap (see Supplementary Fig. 9 and 10). We keep the minimum distance value among those, which we call the nearest neighbor distance, and repeat this process for each cell in P1 to form a distribution of nearest neighbor distances, di. Measures of central tendencies for di were recorded as well as a histogram of frequencies of di using a bin width of 2 pixels up to 120 pixels. A counting error was assigned to each bin as being the square root of the number of entries. The normalization was chosen to be the total number of cells in the sample core, such that the integral of the histogram is equal to the density of the base phenotype being considered.

Population statistics or the average histogram shape were obtained by computing the mean value for each bin given a sub-population sample. The counting error was propagated and summed in quadrature with the standard error of the mean. The combined error is shown in the shaded band. To establish a test of statistical significance between two different histograms, we first define a test statistic as being the summed log-likelihood that each bin in the distribution has the same mean between two sub-populations. The natural log of the p-value, or “likelihood”, from a t-test between the individual bin values is taken. If the hypothesis sub-population mean is larger, this likelihood is defined to be positive, else it is negative. Schematically, large positive likelihoods represent significant upward fluctuations while large negative likelihoods represent significant downward fluctuations. These likelihoods are then summed across all bins. This forms the observed (hypothesis) statistic. The summed log-likelihood was then recomputed for 1k iterations using randomly assigned sub-populations which have the same number of patients as the test sub-population. This forms the null distribution for the test statistic. The final p-value reported is the one-sided integral of the resultant null distribution from the observed value (see Supplementary Fig. 10). This method overestimates the p-value since real differences in the sub-population can be double-counted when building the null distribution. However, this method treats bin-to-bin correlations correctly since it samples from real data.

Patient baseline characteristics and disease factors were summarized using descriptive statistics. Categorical variables were compared using the two-sided Pearson χ^2 test. A comparison of IHC scoring was performed by a two-sided t-test and plotted as previously described 77. Univariate and multivariate Cox proportional-hazards model was used to test the independent and combined prognostic values of proteins of interest with/without the presence of selected clinical variables. Spearman rank correlations were used to assess the relationship between protein H-score and gene expression (RPKM) values 78. The significance of individual hazard ratios was estimated by Wald’s test. Optimal cut-off points for H-score were determined as previously described 6,79 (see Supplementary Fig. 3). The solid lines and histogram present data for samples with levels higher (red) or lower (blue); the dashed lines present data for samples divided into two groups (higher-red or lower-blue) based on the "optimal cut-off" algorithm 79. Unsupervised hierarchical clustering of IHC protein score from all breast cancer samples was performed using complete linkage and distance correlations with the number of bootstrap replications (n=1000) using the ‘pvclust’ R package 80. The estimated clustering stability is measured by AU (approximately unbiased) (red) p-value and BP (bootstrap probability) (green) value for each cluster in a dendrogram 80 (see Supplementary Fig. 12). To explore the expression value together with clinical-pathological information, a heatmap was drawn where patients were arranged based on the order of the hierarchical clustering outcome.

Genetic admixture analysis For admixture analysis, RNA-Seq reads from 136 breast cancer patients were aligned to hg19 using STAR v2.5.2b 85 with subsequent variant calling completed using GATK (v3.8) HaplotypeCaller 54,86. After variant calling, Admixture v1.3.0 55 was used to estimate ancestry proportions based on reference populations from the 1000 Genomes Project phase 3 87 super populations. Rare variants (i.e., <5% across all phase 3 1000 genomes), all INDELs, and any SNPs that were not biallelic were removed before analysis.

For manuscripts utilizing custom algorithms or software that are central to the research but not yet described in published literature, software must be made available to editors/reviewers. We strongly encourage code deposition in a community repository (e.g. GitHub). See the Nature Research guidelines for submitting code & software for further information.

Data

Policy information about availability of data

All manuscripts must include a data availability statement. This statement should provide the following information, where applicable:

- Accession codes, unique identifiers, or web links for publicly available datasets
- A list of figures that have associated raw data
- A description of any restrictions on data availability

Provide your data availability statement here.

Field-specific reporting

Please select the one below that is the best fit for your research. If you are not sure, read the appropriate sections before making your selection.

[x] Life sciences [ ] Behavioural & social sciences [ ] Ecological, evolutionary & environmental sciences

For a reference copy of the document with all sections, see nature.com/documents/nr-reporting-summary-flat.pdf

Life sciences study design

All studies must disclose on these points even when the disclosure is negative.

Sample size	Patient samples were collected from all available samples with complete clinicopathological data in cancer registry and histological data
Data exclusions	Patients samples with incomplete or missing information were excluded from the analysis. All experimental data is the result of multiple independent experiments as detailed in figure legends, not data from these experiments were excluded.
Replication	Reproducibility was confirmed by performing independent biological replicates as detailed in the figure legends.
Randomization	Patient stratification in this retrospective study was based on patient and clinicopathologic features. No procedure were used in which randomization was required.
Blinding	Investigators were not blinded during data collection however data was digitally analyzed in large aggregates using identifiers that gave no indication of patient features or group allocation prior to analysis. Given readily observable scale of differences observed in experiments with cell lines investigators were not blinded.

Reporting for specific materials, systems and methods

We require information from authors about some types of materials, experimental systems and methods used in many studies. Here, indicate whether each material, system or method listed is relevant to your study. If you are not sure if a list item applies to your research, read the appropriate section before selecting a response.

Materials & experimental systems

n/a	Involved in the study	
[ ]	[x]	Antibodies
[ ]	[x]	Eukaryotic cell lines
[x]	[ ]	Palaeontology
[x]	[ ]	Animals and other organisms
[ ]	[x]	Human research participants
[x]	[ ]	Clinical data

Methods

n/a	Involved in the study	
[x]	[ ]	ChIP-seq
[x]	[ ]	Flow cytometry
[x]	[ ]	MRI-based neuroimaging

Antibodies

Antibodies used

Ab Dil Vendor Clone Catalog #
ERa 1:35 Dako 1D5 MA5-13191
PR 1:50 Dako PgR 636 MA5-12581

	HER2 (RTU) DAKO A0485<br>E-Cad 1:50 Dako M361201-2<br>EGFR 1:500 Dako DAK-H1-WT<br>GATA3 1:50 Santa Cruz HG3-31<br>Kaiso 1:1,000 Abcam (6F) ab12723
Validation	Validation data is available at the manufacterer's websites with published reference and application specific data provided. No new antibodies were used in this study.

Eukaryotic cell lines

Policy information about cell lines

Cell line source(s)	MDA-MB-231 were from ATCC
Authentication	Cells line purchased and constructed were authenticated using STR profiling
Mycoplasma contamination	All cell lines were tested for mycobacteria and determined to be negative.
Commonly misidentified lines<br>(See ICLAC register)	No commonly misidentified cell lines were used in this study

Human research participants

Policy information about studies involving human research participants

Population characteristics	Following IRB approval from East Carolina University and the National Institutes of Health intramural research program, de-identified formalin-fixed and paraffin-embedded (FFPE) tissue samples and de-identified clinical information abstracted from the medical records were requisitioned and initially procured for 733 breast cancer patients who underwent surgery for Stage 0 to Stage IV breast cancer between 2001 and 2010 at Pitt County Memorial Hospital (now Vidant Medical Center), Greenville, NC. Race, ethnicity, or “ancestry” was self-reported at the initial visit and captured in the medical record. Survival was recorded retrospectively from the medical records and the cancer registry.
Recruitment	All patient samples and data obtained were de-identified and approved by the East Carolina University Institutional Review Board as a human subject exempt project, for which no informed consent is needed. The study was conducted in accordance with the Declaration of Helsinki.
Ethics oversight	Following IRB approval from East Carolina University and the National Institutes of Health intramural research program, de-identified formalin-fixed and paraffin-embedded (FFPE) tissue samples and de-identified clinical information abstracted from the medical records were requisitioned and initially procured for 733 breast cancer patients who underwent surgery for Stage 0 to Stage IV breast cancer between 2001 and 2010 at Pitt County Memorial Hospital (now Vidant Medical Center), Greenville, NC. Race, ethnicity, or “ancestry” was self-reported at the initial visit and captured in the medical record. Survival was recorded retrospectively from the medical records and the cancer registry.

Note that full information on the approval of the study protocol must also be provided in the manuscript.
